# A Novel Approach to the Partial Information Decomposition

**DOI:** 10.3390/e24030403

**Published:** 2022-03-13

**Authors:** Artemy Kolchinsky

**Affiliations:** Santa Fe Institute, Santa Fe, NM 87501, USA; artemyk@gmail.com

**Keywords:** partial information decomposition, redundancy, synergy

## Abstract

We consider the “partial information decomposition” (PID) problem, which aims to decompose the information that a set of source random variables provide about a target random variable into separate redundant, synergistic, union, and unique components. In the first part of this paper, we propose a general framework for constructing a multivariate PID. Our framework is defined in terms of a formal analogy with intersection and union from set theory, along with an ordering relation which specifies when one information source is more informative than another. Our definitions are algebraically and axiomatically motivated, and can be generalized to domains beyond Shannon information theory (such as algorithmic information theory and quantum information theory). In the second part of this paper, we use our general framework to define a PID in terms of the well-known Blackwell order, which has a fundamental operational interpretation. We demonstrate our approach on numerous examples and show that it overcomes many drawbacks associated with previous proposals.

## 1. Introduction

Understanding how information is distributed in multivariate systems is an important problem in many scientific fields. In the context of neuroscience, for example, one may wish to understand how information about an external stimulus is encoded in the activity of different brain regions. In computer science, one might wish to understand how the output of a logic gate reflects the information present in different inputs to that gate. Numerous other examples abound in biology, physics, machine learning, cryptography, and other fields [1,2,3,4,5,6,7,8,9,10].

Formally, suppose that we are provided with a random variable *Y* which we call the “target”, as well as a set of *n* random variables $X_{1},\ldots ,X_{n}$ which we call the “sources”. The *partial information decomposition* (PID), first proposed by Williams and Beer in 2010 [11], aims to quantify how information about the target is distributed among the different sources. In particular, the PID seeks to decompose the mutual information provided jointly by all sources into a set of nonnegative terms, such as *redundancy* (information present in each individual source), *synergy* (information only provided by the sources jointly, not individually), *union information* (information provided by at least one individual source), and *unique information* (information provided by only one individual source).

As discussed in detail below, the PID is inspired by an analogy between information theory and set theory. In this analogy, the information that the sources provide about the target are imagined as sets, while PID terms such as redundancy, union information, and synergy are imagined as the sizes of intersections, unions, and complements. While the analogy between information-theoretic and set-theoretic quantities is suggestive, it does not specify how to actually define the PID. Moreover, it has also been shown that existing measures from information theory (such as mutual information and conditional mutual information) cannot be used directly to construct the PID, since these measures conflate contributions from different terms like synergy and redundancy [11,12]. In response, many proposals for how to define PID terms have been advanced [5,13,14,15,16,17,18,19,20,21]. However, existing proposals suffer from various drawbacks, such as behaving counterintuitively on simple examples, being limited to only two sources, or lacking a clear operational interpretation. Today there is no generally agreed-upon way of defining the PID.

In this paper, we propose a new and principled approach to the PID which addresses these drawbacks. Our approach can handle any number of sources and can be justified in algebraic, axiomatic, and operational terms. We present our approach in two parts.

In part I (Section 4), we propose a general framework for defining the PID. Our framework does not prescribe specific definitions, but instead shows how an information-theoretic decomposition can be grounded in a formal analogy with set theory. Specifically, we consider the definitions of “set intersection” and “set union” in set theory: the intersection of sets $S_{1},S_{2},\ldots$ is the largest set that is contained in all of the $S_{i}$, while the union of sets $S_{1},S_{2},\ldots$ is the smallest set that contains all of the $S_{i}$. As we show, these set-theoretic definitions can be mapped into information-theoretic terms by treating “sets” as random variables, “set size” as mutual information between a random variable and the target *Y*, and “set inclusion” as some externally specified ordering relation ⊏, which specifies when one random variable is more informative than another. Using this mapping, we define information-theoretic redundancy and union information in the same way that the sizes of intersections and unions are defined in set theory (other PID terms, such as synergy and unique information, can be computed in a straightforward way from redundancy and union information). Moreover, while our approach is motivated by set-theoretic intuitions, as we show in Section 4.2, it can also be derived from an alternative axiomatic foundation. We finish part I by reviewing relevant prior work in information theory and the PID literature. We also discuss how our framework can be generalized beyond the standard setting of the PID and even beyond Shannon information theory, to domains like algorithmic information theory and quantum information theory.

One unusual aspect of our framework is that it provides independent definitions of union information and redundancy. Most prior work on the PID has focused exclusively on the definition of redundancy, because it assumed that union information can be determined from redundancy using the so-called “inclusion-exclusion principle”. In Section 4.3, we argue that the inclusion-exclusion principle should not be expected to hold in the context of the PID.

Part I provides a general framework. Concrete definitions of the PID can be derived from this general framework by choosing a specific “more informative” ordering relation ⊏. In fact, the study of ordering relations between information sources has a long history in statistics and information theory [22,23,24,25,26,27]. One particularly important relation is the so-called “Blackwell order” [13,28], which has a fundamental operational interpretation in terms of utility maximization in decision theory.

In part II of this paper (Section 5), we combine the general framework developed in part I with the Blackwell order. This gives rise to concrete definitions of redundancy and union information. We show that our measures behave intuitively and have simple operational interpretations in terms of decision theory. Interestingly, while our measure of redundancy is novel, our measure of union information has previously appeared in the literature under a different guise [13,17].

In Section 6, we compare our redundancy measure to previous proposals, and illustrate it with various bivariate and multivariate examples. We finish the paper with a discussion and proposals for future work in Section 7.

We introduce some necessary notation and preliminaries in the next section. In addition, we provide background regarding the PID in Section 3. All proofs, as well as some additional results, are found in the appendix.

## 2. Notation and Preliminaries

We use uppercase letters (Y,X,Q,…) to indicate random variables over some underlying probability space. We use lowercase letters (y,x,q,…) to indicate specific outcomes of random variables, and calligraphic letters (Y,X,Q…) to indicate sets of outcomes. We often index random variables with a subscript, e.g., the random variable $X_{i}$ with outcomes $x_{i}\in X_{i}$ (so $x_{i}$ does not refer to the $i^{\mathrm{th}}$ outcome of random variable *X*, but rather to some generic outcome of random variable $X_{i}$). We use notation like A−B−C to indicate that *A* is conditionally independent of *C* given *B*. Except where otherwise noted, we assume that all random variables have a finite number of outcomes.

We use notation like $P_{X}\left(x\right)$ to indicate the probability distribution associated with random variable *X*, $P_{XY}\left(x,y\right)$ to indicate the joint probability distribution associated with random variables *X* and *Y*, and $P_{X|Y}\left(x|y\right)$ to indicate the conditional probability distribution of *X* given *Y*. Given two random variables *X* and *Y* with outcome sets X and Y, we use notation like $\kappa_{X|Y}\left(x|y\right)$ to indicate some stochastic *channel* of outputs x∈X given inputs y∈Y. In general, a channel $\kappa_{X|Y}$ specifies some arbitrary conditional distribution of *X* given *Y*, which can be different from $P_{X|Y}$, the actual conditional distribution of *X* given *Y* (as determined by the underlying probability space).

As described above, we consider the information that a set of “source” random variables $X_{1},\ldots ,X_{n}$ provide a “target” random variable *Y*. Without loss of generality, we assume that the marginal distributions $P_{Y}$ and $P_{X_{i}}$ for all *i* have full support (if they do not, one can restrict Y and/or $X_{i}$ to outcomes that have strictly positive probability).

Finally, note that despite our use of the terms “source” and “target”, we do not assume any causal directionality between the sources and target (see also discussion in [29]). For example, in neuroscience, *Y* might be an external stimulus which causes the activity of brain regions $X_{1},\ldots ,X_{n}$, while in computer science *Y* might represent the output of a logic gate caused by inputs $X_{1},\ldots ,X_{n}$ (so the causal direction is reversed). In yet other contexts, there could be other causal relationships among $X_{1},\ldots ,X_{n}$ and *Y*, or they might not be causally related at all.

## 3. Background on the Partial Information Decomposition (PID)

Given a set of sources $X_{1},\ldots ,X_{n}$ and a target *Y*, the PID aims to decompose $I(Y;X_{1},\ldots ,X_{n})$, the total mutual information provided by all sources about the target, into a set of nonnegative terms such as [11,12]:

*Redundancy* $I_{\cap }\left(X_{1};\ldots ;X_{n}\;\to \;Y\right)$, the information present in each individual source. Redundancy can be considered as the intersection of the information provided by different sources and is sometimes called “intersection information” in the literature [16,18].
*Union information* $I_{\cup }\left(X_{1};\ldots ;X_{n}\;\to \;Y\right)$, the information provided by at least one individual source [12,17].
*Synergy* $S(X_{1};\ldots ;X_{n}\;\to \;Y)$, the information found in the joint outcome of all sources, but not in any of their individual outcomes. Synergy is defined as [17]
  (1) $$\begin{array}{cc} S(X_{1};\ldots ;X_{n}\;\to \;Y) & =I\left(Y;X_{1},\ldots ,X_{n}\right)-I_{\cup }\left(X_{1};\ldots ;X_{n}\;\to \;Y\right). \end{array}$$
*Unique information* in source $X_{i}$, $U(X_{i}\;\to \;Y|X_{1};\ldots ;X_{n})$, the non-redundant information in each particular source. Unique information is defined as
  (2) $$\begin{array}{cc} U(X_{i}\;\to \;Y|X_{1};\ldots ;X_{n}) & =I\left(Y;X_{i}\right)-I_{\cap }\left(X_{1};\ldots ;X_{n}\;\to \;Y\right). \end{array}$$

In addition to the above terms, one can also define *excluded information*,

(3) $$E\left(X_{i}\;\to \;Y|X_{1};\ldots ;X_{n}\right)=I_{\cup }\left(X_{1};\ldots ;X_{n}\;\to \;Y\right)-I\left(Y;X_{i}\right),$$

as the information in the union of the sources which is not in a particular source $X_{i}$. To our knowledge, excluded information has not been previously considered in the PID literature, although it is the natural “dual” of unique information as defined in Equation (2).

**Figure 1:**
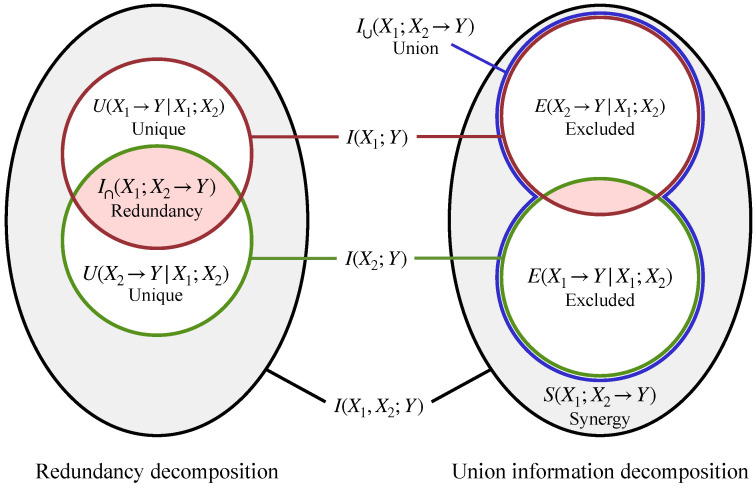
Partial information decomposition of the information provided by two sources about a target. On the left, we show the decomposition induced by redundancy $I_{\cap }$, which leads to measures of unique information *U*. On the right, we show the decomposition induced by union information $I_{\cup }$, which leads to measures of synergy *S* and excluded information *E*.

Given the definitions above, once a measure of redundancy $I_{\cap }$ is chosen, unique information is determined by Equation (2). Similarly, once a measure of union information $I_{\cup }$ is chosen, synergy and excluded information are determined by Equations (1) and (3). In Figure 1, we illustrate the relationships between these different PID terms for the simple case of two sources, $X_{1}$ and $X_{2}$. We show two different decompositions of the information provided by the sources jointly, $I(X_{1},X_{2};Y)$, and individually, $I(X_{1};Y)$ and $I(X_{2};Y)$. The diagram on the left shows the decomposition defined in terms of redundancy $I_{\cap }$, while the diagram on the right shows the decomposition defined in terms of union information $I_{\cup }$.

When more than two sources are present, the PID can be used to define additional terms, beyond the ones shown in Figure 1. For example, for three sources, one can define redundancy terms like $I_{\cap }\left(X_{1},X_{2},X_{3}\;\to \;Y\right)$ (representing the information found in all individual sources) as well as redundancy terms like $I_{\cap }\left(\left(X_{1},X_{2}\right),\left(X_{1},X_{3}\right),\left(X_{2},X_{3}\right)\;\to \;Y\right)$ (representing the information found in all pairs of sources), and similarly for union information.

The idea that redundancy and union information lead to two different information decompositions is rarely discussed in the literature. In fact, the very concept of union information is rarely discussed in the literature explicitly (although it often appears in an implicit form via measures of synergy, since synergy is related to union information through Equation (1)). As we discuss below in Section 4.3, the reason for this omission is that most existing work assumes (whether implicitly or explicitly) that redundancy and union information are not independent measures, but are instead related via the so-called “inclusion-exclusion principle”. If the inclusion-exclusion principle is assumed to hold, then the distinction between the two decompositions disappears. We discuss this issue in greater detail below, where we also argue that the inclusion-exclusion principle should not be expected to hold in the context of the PID.

We have not yet described how the redundancy and union information measures $I_{\cap }$ and $I_{\cup }$ are defined. In fact, this remains an open research question in the field (and one which this paper will address). When they first introduced the idea of the PID, Williams and Beer proposed a set of intuitive axioms that any measure of redundancy should satisfy [11,12], which we summarize in Appendix A. In later work, Griffith and Koch [17] proposed a similar set of axioms that union information should satisfy, which are also summarized in Appendix A. However, these axioms do not uniquely identify a particular measure of redundancy or union information.

Williams and Beer also proposed a particular redundancy measure which satisfies their axioms, which we refer to as $I_{\cap }^{\mathrm{WB}}$ [11,12]. Unfortunately, $I_{\cap }^{\mathrm{WB}}$ has been shown to behave counterintuitively in some simple cases [19,20]. For example, consider the so-called “COPY gate”, where there are two sources $X_{1}$ and $X_{2}$ and the target is a copy of their joint outcomes, $Y=(X_{1},X_{2})$. If $X_{1}$ and $X_{2}$ are statistically independent, $I(X_{1};X_{2})=0$, then intuition suggests that the two sources provide independent information about *Y* and therefore that redundancy should be 0. In general, however, $I_{\cap }^{\mathrm{WB}}\left(X_{1};X_{2}\;\to \;Y\right)$ does not vanish in this case. To avoid this issue, Ince [20] proposed that any valid redundancy measure should obey the following property:

(4) $$\mathrm{If}\;I\left(X_{1};X_{2}\right)=0,\;\mathrm{then}\;I_{\cap }\left(X_{1};X_{2}\;\to \;\left(X_{1},X_{2}\right)\right)=0,$$

which is called the *Independent identity property*.

In recent years, many other redundancy measures have been proposed [13,15,16,18,19,20,21]. However, while some of these proposals satisfy the Independent identity property, they suffer various other drawbacks, such as exhibiting other types of counterintuitive behavior, being limited to two sources, and/or lacking a clear operational motivation. We discuss some of these previously proposed measures in Section 4.4, Section 5.4 and Section 6.

Unlike redundancy, to our knowledge only two measures of union information have been advanced. The first one appeared in the original work on the PID [12], and was derived from $I_{\cap }^{\mathrm{WB}}$ using the inclusion-exclusion principle. The second one appeared more recently [13,17] and is discussed in Section 5.4 below.

## 4. Part I: Redundancy and Union Information from an Ordering Relation

### 4.1. Introduction

As mentioned above, PID is motivated by an informal analogy with set theory [12]. In particular, redundancy is interpreted analogously to the size of the intersection of the sources $X_{1},\ldots ,X_{n}$, while union information is interpreted analogously to the size of their union.

We propose to define the PID by making this analogy formal, and in particular by going back to the algebraic definitions of intersection and union in set theory. In pursuing this direction, we build on a line of previous work in information theory and PID, which we discuss in Section 4.4.

Recall that in set theory, the intersection of sets $S_{1},\ldots ,S_{n}\subseteq U$ (where *U* is some universal set) is the largest set that is contained in all $S_{i}$ (Section 7.2, [30]). This means that the size of the intersection can be written as

(5) $$\begin{array}{cc} \left|\underset{i}{⋂}S_{i}\right| & =\underset{T\subseteq U}{\sup}\;\left|T\right|\;\mathrm{such}\;\mathrm{that}\;\forall i\;T\subseteq S_{i}, \end{array}$$

Similarly, the union of sets $S_{1},\ldots ,S_{n}\subseteq U$ is the smallest set that contains all $S_{i}$ (Section 7.2, [30]), so the size of the union can be written as

(6) $$\begin{array}{cc} \left|\underset{i}{⋃}S_{i}\right| & =\underset{T\subseteq U}{\inf}\;\left|T\right|\;\;\mathrm{such}\;\mathrm{that}\;\forall i\;S_{i}\subseteq T. \end{array}$$

Equations (5) and (6) are useful because they express the size of the intersection and union via an optimization over simpler terms (the size of individual sets, |T|, and the subset inclusion relation, ⊆).

We translate these definitions to the information-theoretic setting of the PID. We take the analogue of a “set” to be some random variable *A* that provides information about the target *Y*, and the analogue of “set size” to be the mutual information I(A;Y). In addition, we assume that there is some ordering relation ⊏ between random variables analogous to set inclusion ⊆. Given such a relation, the expression A⊏B means that random variable *B* is “more informative” than *A*, in the sense that the information that *A* provides about *Y* is contained within the information that *B* provides about *Y*.

At this point, we leave the ordering relation ⊏ unspecified. In general, we believe that the choice of ⊏ will not be determined from purely information-theoretic considerations, but may instead depend on the operational setting and scientific domain in which the PID is applied. At the same time, there has been a great deal of research on ordering relations in statistics and information theory. In part II of this paper, Section 5, we will combine our general framework with a particular ordering relation, the so-called “Blackwell order”, which has a fundamental interpretation in terms of decision theory.

We now provide formal definitions of redundancy and union information, relative to the choice of ordering relation ⊏. In analogy to Equation (5), we define redundancy as

(7) $$\begin{array}{cc} I_{\cap }\left(X_{1};\ldots ;X_{n}\;\to \;Y\right) & :=\underset{Q}{\sup}I\left(Q;Y\right)\;\mathrm{such}\;\mathrm{that}\;\forall i\;Q⊏X_{i} \end{array}$$

where the maximization is over all random variables with a finite number of outcomes. Thus, redundancy $I_{\cap }$ is the maximum information about *Y* in any random variable that is less informative than all of the sources. In analogy with Equation (6), we define union information as

(8) $$\begin{array}{cc} I_{\cup }(X_{1};\ldots ;X_{n} & \;\to \;Y):=\underset{Q}{\inf}I\left(Q;Y\right)\;\mathrm{such}\;\mathrm{that}\;\forall i\;X_{i}⊏Q \end{array}$$

Thus, union information $I_{\cup }$ is the minimum information about *Y* in any random variable that is more informative than all of the sources. Given these definitions, other elements of the PID (such as unique information, synergy, and excluded information) can be defined using the expressions found in Section 3. Note that $I_{\cap }$ and $I_{\cup }$ depend the choice of ordering relation ⊏, although for convenience we leave this dependence implicit in our notation.

One of the attractive aspects of our definitions is that they do not simply quantify the amount of redundancy and union information, but also specify the “content” of that redundant and union information. In particular, the random variable *Q* that achieves the optimum in Equation (7) specifies the content of the redundant information via the joint distribution $P_{YQ}$. Similarly, the random variable *Q* which achieves the optimum in Equation (8) specifies the content of the union information via the joint distribution $P_{YQ}$. Note that these optimizing *Q* may not be unique, reflecting the fact that there may be different ways to represent the redundancy or union information. (Note also that the supremum or infinitum may not be achieved in Equations (7) and (8), in which case one can consider *Q* that achieve the optimal values to any desired precision ϵ>0.)

So far we have not made any assumptions about the ordering relation ⊏. However, we can derive some useful bounds by introducing three weak assumptions:

- Monotonicity of mutual information: A⊏B⇒I(A;Y)≤I(B;Y) (less informative sources have less mutual information).
- Reflexivity: A⊏A for all *A* (each source is at least as informative as itself).
- For all sources $X_{i}$, $O⊏X_{i}⊏\left(X_{1},\ldots ,X_{n}\right)$, where *O* indicates a constant random variable with a single outcome and $\left(X_{1},\ldots ,X_{n}\right)$ indicates all sources considered jointly (each source is more informative than a trivial source and less informative than all sources jointly).

Assumptions I and II imply that the redundancy and union information of a single source are equal to the mutual information in that source:

$$I_{\cap }\left(X_{1}\;\to \;Y\right)=I_{\cup }\left(X_{1}\;\to \;Y\right)=I\left(X_{1};Y\right).$$

Assumptions I and III imply the following bounds on redundancy and union information:

(9) $$\begin{array}{cc} 0 & \leq I_{\cap }\left(X_{1};\ldots ;X_{n}\;\to \;Y\right)\leq \min_{i}I\left(Y;X_{i}\right). \end{array}$$

(10) $$\begin{array}{cc} \max_{i}I\left(Y;X_{i}\right) & \leq I_{\cup }\left(X_{1};\ldots ;X_{n}\;\to \;Y\right)\leq I\left(Y;X_{1},\ldots ,X_{n}\right). \end{array}$$

Equation (9) in turn implies that the unique information in each source $X_{i}$, as defined in Equation (2), is bounded between 0 and $I(Y;X_{i})$. Similarly, Equation (10) implies that the synergy, as defined in Equation (1), obeys

$$0\leq S\left(X_{1};\ldots ;X_{n}\;\to \;Y\right)\leq \min_{i}I\left(Y;X_{1},\ldots ,X_{n}|X_{i}\right),$$

where we have used the chain rule $I\left(Y;X_{1},\ldots ,X_{n}\right)=I\left(Y;X_{i}\right)+I\left(Y;X_{1},\ldots ,X_{n}|X_{i}\right)$. Equation (10) also implies that excluded information in each source $X_{i}$, as defined in Equation (3), is bounded between 0 and $I(Y;X_{1},\ldots ,X_{n}|X_{i})$.

Note that in general, stronger orders give smaller values of redundancy and larger values of union information. Consider two orders ⊏ and ${⊏}^{\prime }$ where the first one is stronger than the second: $A⊏B\Rightarrow A{⊏}^{\prime }B$ for all *A* and *B*. Then, any *Q* in the feasible set of Equation (7) under ⊏ will also be in the feasible set under ${⊏}^{\prime }$, and similarly for Equation (8). Therefore, $I_{\cap }$ defined relative to ⊏ will have a lower value than $I_{\cap }$ defined relative to ${⊏}^{\prime }$, and vice versa for $I_{\cup }$.

In the rest of this section, we discuss alternative axiomatic justifications for our general framework, the role of the inclusion-exclusion principle, relation to prior work, and further generalizations. Readers who are more interested in the use of our framework to define concrete measures of redundancy and union information may skip to Section 5.

### 4.2. Axiomatic Derivation

In Section 4.1, we defined the PID in terms of an algebraic analogy with intersection and union in set theory. This definition can be considered as the primary one in our framework. At the same time, the same definitions can also be derived in an alternative manner from a set of axioms, as commonly sought after in the PID literature. In particular, in Appendix B, we prove the following result regarding redundancy.

**Theorem 1.**  *Any redundancy measure that satisfies the following five axioms is equal to $I_{\cap }\left(X_{1};\ldots ;X_{n}\;\to \;Y\right)$ as defined in Equation *(7)*.*

1. Symmetry: *$I_{\cap }\left(X_{1};\ldots ;X_{n}\;\to \;Y\right)$ is invariant to the permutation of $X_{1},\ldots ,X_{n}$.*
2. Self-redundancy: *$I_{\cap }\left(X_{1}\;\to \;Y\right)=I\left(Y;X_{1}\right)$.*
3. Monotonicity: *$I_{\cap }\left(X_{1};\ldots ;X_{n}\;\to \;Y\right)\leq I_{\cap }\left(X_{1};\ldots ;X_{n-1}\;\to \;Y\right)$.*
4. Order equality: *$I_{\cap }\left(X_{1};\ldots ;X_{n}\;\to \;Y\right)=I_{\cap }\left(X_{1};\ldots ;X_{n-1}\;\to \;Y\right)$ if $X_{i}⊏X_{n}$ for some i<n.*
5. Existence: *There is some Q such that $I_{\cap }\left(X_{1};\ldots ;X_{n}\;\to \;Y\right)=I\left(Y;Q\right)$ and $Q⊏X_{i}$ for all i.*

While *Symmetry*, *Self-redundancy*, and *Monotonicity* axioms are standard in the PID literature (see Appendix A), the last two axioms require some explanation. *Order equality* is a generalization of the previously proposed *Deterministic equality* axiom, described in Appendix A, where the condition $X_{i}=f\left(X_{n}\right)$ (deterministic relationship) is generalized to the “more informative” relation $X_{i}⊏X_{n}$. This axiom reflects the idea that if a new source $X_{n}$ is more informative than an existing source $X_{i}$, then redundancy shouldn’t decrease when $X_{n}$ is added.

*Existence* is the most novel of our proposed axioms. It says that for any set of sources $X_{1},\ldots ,X_{n}$, there exists some random variable which captures the redundant information. It is similar to the statement in axiomatic set theory that the intersection of a collection of sets is itself a set (note that in Zermelo-Fraenkel set theory, this statement is derived from the Axiom of Separation).

We can derive a similar result for union information (proof in Appendix B).

**Theorem 2.**  *Any union information measure that satisfies the following five axioms is equal to $I_{\cup }\left(X_{1};\ldots ;X_{n}\;\to \;Y\right)$ as defined in Equation *(8)*.*

1. Symmetry: *$I_{\cup }\left(X_{1};\ldots ;X_{n}\;\to \;Y\right)$ is invariant to the permutation of $X_{1},\ldots ,X_{n}$.*
2. Self-union: *$I_{\cup }\left(X_{1}\;\to \;Y\right)=I\left(Y;X_{1}\right)$.*
3. Monotonicity: *$I_{\cup }\left(X_{1};\ldots ;X_{n}\;\to \;Y\right)\geq I_{\cup }\left(X_{1};\ldots ;X_{n-1}\;\to \;Y\right)$.*
4. Order equality: *$I_{\cup }\left(X_{1};\ldots ;X_{n}\;\to \;Y\right)=I_{\cup }\left(X_{1};\ldots ;X_{n-1}\;\to \;Y\right)$ if $X_{n}⊏X_{i}$ for some i<n.*
5. Existence: *There is some Q such that $I_{\cup }\left(X_{1};\ldots ;X_{n}\;\to \;Y\right)=I\left(Y;Q\right)$ and $X_{i}⊏Q$ for all i.*

These axioms are dual to the redundancy axioms outlined above. Compared to previously proposed axioms for union information, as described in Appendix A, the most unusual of our axioms is *Existence*. It says that given a set of sources $X_{1},\ldots ,X_{n}$, there exists some random variable which captures the union information. It is similar in spirit to the “Axiom of Union” in axiomatic set theory [31].

Finally, note that for some choices of ⊏, there may not exist measures of redundancy and/or union information that satisfy the axioms in Theorems 1 and 2, in which case these theorems still hold but are trivial. However, even in such “pathological” cases, $I_{\cap }$ and $I_{\cup }$ can still be defined via Equations (7) and (8), as long as ⊏ has a “least informative” and a “most informative” element (e.g., as provided by Assumption III above), so that the feasible sets are not empty. In this sense, the definitions in Equations (7) and (8) are more general than the axiomatic derivations provided by Theorems 1 and 2.

### 4.3. Inclusion-Exclusion Principle

One unusual aspect of our approach is that, unlike most previous work, we propose separate measures of redundancy and union information.

Recall that in set theory, the size of the intersection and the union are not independent of each other, but are instead related by the *inclusion-exclusion principle* (IEP). For example, given any two sets *S* and *T*, the IEP states that the size of the union of *S* and *T* is given by the sum of their individual sizes minus the intersection,

(11) $$\left|S\cup T\right|=\left|S\right|+\left|T\right|-\left|S\cap T\right|.$$

More generally, the IEP relates the sizes of intersection and unions for any number of sets, via the following inclusion-exclusion formulas:

(12) $$\begin{array}{cc} \left|\overset{n}{\underset{i=1}{⋃}}S_{i}\right| & =\sum_{⌀\neq J\subseteq \{1,\ldots ,n\}}{\left(-1\right)}^{\left|J\right|-1}|\underset{i\in J}{⋂}S_{i}|. \end{array}$$

(13) $$\begin{array}{cc} \left|\overset{n}{\underset{i=1}{⋂}}S_{i}\right| & =\sum_{⌀\neq J\subseteq \{1,\ldots ,n\}}{\left(-1\right)}^{\left|J\right|-1}|\underset{i\in J}{⋃}S_{i}|. \end{array}$$

Historically, the IEP has played an important role in analogies between set theory and information theory, which began to be explored in 1950s and 1960s [32,33,34,35,36]. Recall that the entropy H(X) quantifies the amount of information gained by learning the outcome of random variable *X*. It has been observed that, for a set of random variables $X_{1},\ldots ,X_{n}$, the joint entropy $H(X_{1},\ldots ,X_{n})$ behaves somewhat like the size of the union of the information in the individual variables. For instance, like the size of the union, joint entropy is subadditive ($H\left(X_{1}\right)+H\left(X_{2}\right)\geq H\left(X_{1},X_{2}\right)$) and increases with additional random variables ($H\left(X_{1},X_{2}\right)\geq H\left(X_{1}\right)$). Moreover, for two random variables $X_{1}$ and $X_{2}$, the mutual information $I\left(X_{1};X_{2}\right)=H\left(X_{1}\right)+H\left(X_{2}\right)-H\left(X_{1},X_{2}\right)$ acts like the size of the intersection of the information provided by $X_{1}$ and $X_{2}$, once intersection is defined analogously to the IEP expression in Equation (11) [35,36]. Given the general IEP formula in Equation (13), this can be used to define the size of the intersection between any number of random variables. For instance, the size of a three-way intersection is

$$\begin{array}{cc} I(X_{1};X_{2};X_{3})= & H(X_{1})+H\left(X_{2}\right)+H\left(X_{3}\right)\\ & \;-H\left(X_{1},X_{2}\right)-H\left(X_{1},X_{3}\right)-H\left(X_{2},X_{3}\right)+H\left(X_{1},X_{2},X_{3}\right), \end{array}$$

a quantity called *co-information* or *interaction information* in the literature [32,33,35,36,37].

Unfortunately, interaction information, as well as other higher-order interaction terms defined via the IEP, can take negative values [32,35,37]. This conflicts with the intuition that information measures should always be non-negative, in the same way that set size is always non-negative.

One of the primary motivations for the PID, as originally proposed by Williams and Beer [11,12], was to solve the problem of negativity encountered by interaction information. To develop a non-negative information decomposition, Williams and Beer took two steps. First, they considered the information that a set of sources $X_{1},\ldots ,X_{n}$ provide about some target random variable *Y*. Second, they developed a non-negative measure of redundancy ($I_{\cap }^{\mathrm{WB}}$) which leads to a non-negative union information once an IEP formula like Equation (12) is applied (Theorem 4.7, [12]). For example, in the original proposal, union information and redundancy are related via

(14) $$I_{\cup }\left(X_{1};X_{2}\;\to \;Y\right)\overset{?}{=}I\left(Y;X_{1}\right)+I\left(Y;X_{2}\right)-I_{\cap }\left(X_{1};X_{2}\;\to \;Y\right),$$

which is the analogue of Equation (11). This can be plugged into expressions like Equation (1), so as to express synergy in terms of redundancy as

(15) $$S\left(X_{1};\ldots ;X_{n}\;\to \;Y\right)\overset{?}{=}I\left(Y;X_{1},\ldots ,X_{n}\right)-I\left(Y;X_{1}\right)-I\left(Y;X_{2}\right)+I_{\cap }\left(X_{1};X_{2}\;\to \;Y\right).$$

The meaning of IEP-based identities such as Equations (14) and (15) can be illustrated using the Venn diagrams in Figure 1. In particular, they imply that the pink region in the right diagram is equal in size to the pink region in the left diagram, and that the grey region in the left diagram is equal in size to the grey region in the right diagram. More generally, IEP implies an equivalence between the information decomposition based on redundancy and the one based on union information.

As mentioned in Section 3, due to shortcomings in the original redundancy measure $I_{\cap }^{\mathrm{WB}}$, numerous other proposals for the PID have been advanced. Most of these proposals introduce new measures of redundancy, while keeping the general structure of the PID as introduced by Williams and Beer. In particular, most of these proposals assume that the IEP holds, so that union information can be derived from a measure of redundancy. While the assumption of the IEP is sometimes stated explicitly, more frequently it is implicit in the definitions used. For example, many proposals assume that synergy is related to redundancy via an expression like Equation (15), although (as shown above) this implicitly assumes that the IEP holds. In general, the IEP has been largely an unchallenged and unexamined assumption in the PID field. It is easy to see the appeal of the IEP: it builds on deep-seated intuitions about intersection/union from set theory and Venn diagrams, it has a long history in the information-theoretic literature, and it simplifies the problem of defining the PID since it only requires a measure of redundancy to be defined—rather than a measure of redundancy and a measure of union information. (Note that one can also start from union information and then derive redundancy via the IEP formula in Equation (13), as in Appendix B of Ref. [17], although this is much less common in the literature.)

However, there is a different way to define a non-negative PID, which is still grounded in a formal analogy with set theory but does not assume the IEP. Here, one defines measures of redundancy and union information based on the underlying algebra of intersection and union: the intersection of $X_{1},\ldots ,X_{n}$ is the largest element that is less than each $X_{i}$, while the union is the smallest element that is greater than each $X_{i}$. Given these definitions, intersections and unions are not necessarily related to each numerically, as in the IEP, but are instead related by an algebraic duality.

This latter approach is the one we pursue in our definitions (it has also appeared in some prior work, which we review in the next subsection). In general, the IEP will not hold for redundancy and union information as defined in Equations (7) and (8). (To emphasize this point, we put a question mark in Equations (14) and (15), and made the sizes of the pink and grey regions visibly different in Figure 1). However, given the algebraic and axiomatic justifications for $I_{\cap }$ and $I_{\cup }$, we do not see the violation of the IEP as a fatal issue. In fact, there are many domains where generalizations of intersections and unions do not obey the IEP. For example, it is well-known that the IEP is violated in the domain of vector spaces, once the size of a vector space is measured in terms of its dimension [38]. The PID is simply another domain where the IEP should not be expected to hold.

We believe that many problems encountered in previous work on the PID—such as the failure of certain redundancy measures to generalize to more than two sources, or the appearance of uninterpretable negative synergy values—are artifacts of the IEP assumption. In fact, the following result shows that any measures of redundancy and union information which satisfy several reasonable assumptions must violate the IEP as soon as 3 or more sources are present (the proof, in Appendix I, is based on a construction from [39,40]).

**Lemma 1.**  *Let $I_{\cap }$ be any nonnegative redundancy measure which obeys* Symmetry, Self-redundancy, Monotonicity, *and* Independent identity. *Let $I_{\cup }$ be any union information measure which obeys $I_{\cup }\left(X_{1};\ldots ;X_{n}\;\to \;Y\right)\leq I\left(Y;X_{1},\ldots ,X_{n}\right)$. Then, $I_{\cap }$ and $I_{\cup }$ cannot be related by the inclusion-exclusion principle for 3 or more sources.*

The idea that different information decompositions may arise from redundancy versus synergy (and therefore union information) has recently appeared in the PID literature [15,40,41,42,43]. In particular, Chicharro and Panzeri proposed a PID that involves two decomposition: an “information gain” decomposition based on redundancy and an “information loss” decomposition based on synergy [41]. These decompositions correspond to the two Venn diagrams shown in Figure 1.

### 4.4. Relation to Prior Work

Here we discuss prior work which is relevant to our algebraic approach to the PID.

First, note that our definitions of redundancy and union information in Equations (7) and (8) are closely related to notions of “meet” and “join” in a field of algebra called order theory, which generalize intersections and unions to domains beyond set theory [44]. Given a set of objects *S* and an order ⊏, the *meet* of a,b∈S is the unique largest c∈S that is smaller than both *a* and *b*: c⊏a,c⊏b and d⊏c for any *d* that obeys d⊏a,d⊏b. Similarly, the *join* of a,b∈S is the unique smallest *c* that is larger than both *a* and *b*: a⊏c,a⊏c and c⊏d for any *d* that obeys a⊏d,b⊏d. Note that meets and joins are only defined when ⊏ is a special type of partial order called a *lattice*. This is a strict requirement, and many important ordering relations in information theory are not lattices (this includes the “Blackwell order”, which we will consider in part II of this paper [45]).

In our approach, we do not require the ordering relation ⊏ to be a lattice, or even a partial order. We do not require these properties because we do not aim to find the unique union random variable or the unique redundancy random variable. Instead, we aim to quantify the *size of the intersection* and *the size of the union*, which we do by optimizing mutual information subject to constraints, as Equations (7) and (8). These definitions are well-defined even when ⊏ is not a lattice, which allows us to consider a much broader set of ordering relations.

We mention three important precursors of our approach that have been proposed in the PID literature. First, Griffith et al. [16] considered the following order between random variables:

(16) A⊲B iff A=f(B) for some deterministic function f.

This ordering relation ⊲ was first considered in a 1953 paper by Shannon [22], who showed that it defines a lattice over random variables. That paper was the first to introduce the algebraic idea of meets and joins into information theory, leading to an important line of subsequent research [46,47,48,49,50]. Using this order, Ref. [16] defined redundancy as the maximum mutual information in any random variable that is a deterministic function of all of the sources,

(17) $$I_{\cap }^{⊲}\left(X_{1};\ldots ;X_{n}\;\to \;Y\right):=\max_{Q}I\left(Q;Y\right)\;\;\mathrm{such}\;\mathrm{that}\;\;\forall i\;Q⊲X_{i},$$

which is clearly a special case of Equation (7). Unfortunately, in practice, $I_{\cap }^{⊲}$ is not a useful redundancy measure, as it tends to give very small values and is highly discontinuous. For example, $I_{\cap }^{⊲}\left(X_{1};\ldots ;X_{n}\;\to \;Y\right)=0$ whenever the joint distribution $P_{X_{1}\ldots X_{n}Y}$ has full support, meaning that it vanishes on almost all joint distributions [16,18,47]. The reason for this counterintuitive behavior is that the order ⊲ formalizes an extremely strict notion of “more informative”, which is not robust to noise.

Given the deficiencies of $I_{\cap }^{⊲}$, Griffith and Ho [18] proposed another measure of redundancy (also discussed as $I_{\cap }^{2}$ in Ref. [49]),

(18) $$I_{\cap }^{\mathrm{GH}}\left(X_{1};\ldots ;X_{n}\;\to \;Y\right):=\max_{Q}I\left(Q;Y\right)\;\;\mathrm{such}\;\mathrm{that}\;\;\forall i\;Q-X_{i}-Y.$$

This measure is also a special case of Equation (7), where the more informative relation A⊏B is formalized via the conditional independence condition A−B−Y. This measure is similar to the redundancy measure we propose in part II of this paper, and we discuss it in more detail in Section 5.4. (Note that there are some incorrect claims about $I_{\cap }^{\mathrm{GH}}$ in the literature: Lemmas 6 and 7 of Ref. [49] incorrectly state that $I_{\cap }^{\mathrm{GH}}\left(X_{1};X_{2}\;\to \;Y\right)=0$ whenever $X_{1}$ and $X_{2}$ are independent—see the AND gate counterexample in Section 6—while Ref. [18] incorrectly states that $I_{\cap }^{\mathrm{GH}}$ obeys a property called *Target Monotonicity*).

Finally, we mention the so-called “minimum mutual information” redundancy $I_{\cap }^{\mathrm{MMI}}$ [51]. This is perhaps the simplest redundancy measure, being equal to the minimal mutual information in any source: $I_{\cap }^{\mathrm{MMI}}\left(X_{1};\ldots ;X_{n}\;\to \;Y\right):=\min_{i}I\left(X_{i};Y\right)$. It can be written in the form of Equation (7) as

(19) $$I_{\cap }^{\mathrm{MMI}}\left(X_{1};\ldots ;X_{n}\;\to \;Y\right):=\max_{Q}I\left(Q;Y\right)\;\;\mathrm{such}\;\mathrm{that}\;\;\forall i\;I\left(Q;Y\right)\leq I\left(X_{i};Y\right).$$

This redundancy measure has been criticized for depending only on the amount of information provided by the different sources, being completely insensitive to the content of that information. Nonetheless, $I_{\cap }^{\mathrm{MMI}}$ can be useful in some settings, and it plays an important role in the context of Gaussian random variables [51].

Interestingly, unlike $I_{\cap }^{\mathrm{MMI}}$, the original redundancy measure proposed by Williams and Beer [11], $I_{\cap }^{\mathrm{WB}}$, does not appear to be a special case of Equation (7) (at least not under the natural definition of the ordering relation ⊏). We demonstrate this using a counter-example in Appendix H.

As mentioned in Section 4.1, stronger ordering relations give smaller values of redundancy. For the orders considered above, it is easy to show that

(20) A⊲B⇒A−B−Y⇒I(A;Y)≤I(B;Y).

This implies that $I_{\cap }^{⊲}\leq I_{\cap }^{\mathrm{GH}}\leq I_{\cap }^{\mathrm{MMI}}$. In fact, $I_{\cap }^{\mathrm{MMI}}$ is the largest measure that is compatible with the monotonicity of mutual information (Assumption I in Section 4.1).

### 4.5. Further Generalizations

We finish part I of this paper by noting that one can further generalize our approach, by considering other analogues of “set”, “set size”, and “set inclusion” beyond the ones considered in Section 4.1. Such generalizations allow one to analyze notions of information intersection and union in a wide variety of domains, including setups different from the standard one considered in the PID, and domains not based on Shannon information theory.

At a general level, consider a set of object Ω that represents possible “sources”, which may be random variables, as in Section 4.1, or otherwise. Assume there is some function ϕ:Ω→R that quantifies the “amount of information” in a given source Ω, and some relation ⊏ on Ω that indicates which sources are more informative than others. Then, in analogy to Equations (5) and (6), for any set of sources $\{b_{1},\ldots ,b_{n}\}\subseteq \Omega$, one can define redundancy and union information as

(21) $$\begin{array}{cc} I_{\cap }\left(b_{1};\ldots ;b_{n}\right) & :=\underset{a\in \Omega }{\sup}\;\phi \left(a\right)\;\;\mathrm{such}\;\mathrm{that}\;\;\forall i\;a⊏b_{i} \end{array}$$

(22) $$\begin{array}{cc} I_{\cup }\left(b_{1};\ldots ;b_{n}\right) & :=\underset{a\in \Omega }{\inf}\;\phi \left(a\right)\;\;\mathrm{such}\;\mathrm{that}\;\;\forall i\;b_{i}⊏a. \end{array}$$

Synergy, unique, and excluded information can then be defined via Equations (1) to (3).

There are many possible examples of such generalizations, of which we mention a few as illustrations.

*Shannon information theory (beyond mutual information)*. In Section 4.1, ϕ was the mutual information between each random variable and some target *Y*. This can be generalized by choosing a different “amount of information” function ϕ, so that redundancy and union information are quantified in terms of other measures of statistical dependence. Among many other options, possible choices of ϕ include Pearson’s correlation (for continuous random variables) and measures of statistical dependency based *f*-divergences [52], Bregman divergences [53], and Fisher information [54].
*Shannon information theory (without a fixed target)*. The PID can also be defined for a different setup than the typical one considered in the literature. For example, consider a situation where the sources are channels $\kappa_{X_{1}|Y},\ldots ,\kappa_{X_{n}|Y}$, while the marginal distribution over the target *Y* is left unspecified. Here one may take Ω as the set of channels, ϕ as the channel capacity $\phi \left(\kappa_{A|Y}\right):=\max_{P_{Y}}I_{P_{Y}\kappa_{A|Y}}\left(A;Y\right)$, and ⊏ as some ordering relation on channels [24]
*Algorithmic information theory.* The PID can be defined for other notions of information, such as the ones used in Algorithmic Information Theory (AIT) [55]. In AIT, “information” is not defined in terms of statistical uncertainty, but rather in terms of the program length necessary to generate strings. For example, one may take Ω as the set of finite strings, ⊏ as algorithmic conditional independence (a⊏b iff K(y|b)−K(y|b,a)≤const, where K(·|·) is conditional Kolmogorov complexity), and ϕ(a):=K(y)−K(y|a) as the “algorithmic mutual information” with some target string *y*. (This setup is closely related to the notion of algorithmic “common information” [47]).
  *Quantum information theory*. As a final example, the PID can be defined in the context of quantum information theory. For example, one may take Ω as the set of quantum channels, ⊏ as quantum Blackwell order [56,57,58], and ϕ(Φ)=I(ρ,Φ), where I is the Ohya mutual information for some target density matrix ρ under channel Φ∈Ω [59].

## 5. Part II: Blackwell Redundancy and Union Information

In the first part of this paper, we proposed a general framework for defining PID terms. In this section, which forms part II of this paper, we develop a concrete definition of redundancy and union information by combining our general framework with a particular ordering relation ⊏. This ordering relation is called the “Blackwell order”, and it plays a fundamental role in statistics and decision theory [28,45,60]. We first introduce the Blackwell order, then use it to define measures of redundancy and union information, and finally discuss various properties of our measures.

### 5.1. The Blackwell Order

We begin by introducing the ordering relation that we use to define our PID. Given three random variables B,C and *Y*, the ordering relation $B{≺}_{Y}C$ is defined as follows:

(23) $$\begin{array}{cc} B{≺}_{Y}C\;\mathrm{iff}\;P_{B|Y}\left(b|y\right) & =\sum_{c}\kappa_{B|C}\left(b|c\right)P_{C|Y}\left(c|y\right)\;\mathrm{for}\;\mathrm{some}\;\mathrm{channel}\;\kappa_{B|C}\;\mathrm{and}\;\mathrm{all}\;b,y. \end{array}$$

We refer to the relation ${≺}_{Y}$ as the *Blackwell order* relative to random variable *Y*. (Note that the Blackwell order and Blackwell’s Theorem are usually formulated in terms of channels—that is, conditional distributions like $\kappa_{B|Y}$ and $\kappa_{C|Y}$—rather than of random variables as done here. However, these two formulations are equivalent, as shown in [45]).

In words, Equation (23) means the conditional distribution by $P_{B|Y}$ can be generated by first sampling from the conditional distribution $P_{C|Y}$, and then applying some channel $\kappa_{B|C}$ to the outcome. The relation $B{≺}_{Y}C$ implies that $P_{B|Y}$ is more noisy than $P_{C|Y}$ and, by the “data processing inequality” [61], *B* must have less mutual information about *Y* than *C*:

(24) $$\begin{array}{cc} B & {≺}_{Y}C\Rightarrow I\left(B;Y\right)\leq I\left(C;Y\right). \end{array}$$

Intuition suggests that when $B{≺}_{Y}C$, the information that *B* provides about *Y* is contained in the information that *C* provides about *Y*. This intuition is formalized within a decision-theoretic framework using the so-called Blackwell’s Theorem [28,45,60]. To introduce this theorem, imagine a scenario in which *Y* represents the state of the environment. Imagine also that there is an agent who acquires information about the environment via the conditional distribution $P_{B|Y}\left(b|y\right)$, and then uses outcome B=b to select actions a∈A according to some “decision rule” given by the channel $\kappa_{A|B}$. Finally, the agent gains utility according to some utility function u(a,y), which depends on the agent’s action *a* and the environment’s state *y*. The maximum expected utility achievable by any decision rule is given by

(25) $$V_{Y}^{\max}\left(B,u\right):=\max_{\kappa_{A|B}}\sum_{y,b,a}P_{Y}\left(y\right)P_{B|Y}\left(b|y\right)\kappa_{A|B}\left(a|b\right)u\left(a,y\right).$$

From an operational perspective, it is natural to say that *B* is less informative than *C* about *Y* if there is no utility function such that an agent with access to *B* can achieve higher expected utility than an agent with access to *C*. Blackwell’s Theorem states that this is precisely the case if and only if $B{≺}_{Y}C$ [28,45]:

(26) $$B{≺}_{Y}C\;\;\mathrm{iff}\;\;V_{Y}^{\max}\left(B,u\right)\leq V_{Y}^{\max}\left(C,u\right)\;\;\mathrm{for}\;\mathrm{all}\;u.$$

In some sense, this operational description of the relation ${≺}_{Y}$ is deeper than the data processing inequality, Equation (24), which says that $B{≺}_{Y}C$ is sufficient (but not necessary) for I(B;Y)≤I(C;Y). In fact, it can happen that I(B;Y)≤I(C;Y) even though $B{⊀}_{Y}C$ [26,60,62].

A connection between PID and Blackwell’s theorem was first proposed in [13], which argued that the PID should be defined in an operational manner (see Section 5.3 for further discussion of [13]).

### 5.2. Blackwell Redundancy

We now define a measure of redundancy based on the Blackwell order. Specifically, we use our general definition of redundancy, Equation (7), while using the Blackwell order relative to *Y* as the “more informative” relation ⊏:

(27) $$I_{\cap }^{≺}\left(X_{1};\ldots ;X_{n}\;\to \;Y\right):=\underset{Q}{\sup}\;I\left(Q;Y\right)\;\;\mathrm{such}\;\mathrm{that}\;\;\forall i\;Q{≺}_{Y}X_{i}.$$

We refer to this measure as *Blackwell redundancy*.

Given Blackwell’s Theorem, $I_{\cap }^{≺}$ has a simple operational interpretation. Imagine two agents, Alice and Bob, who can acquire information about *Y* via different random variables, and then use this information to maximize their expected utility. Suppose that Alice has access to one of the sources $X_{i}$. Then, the Blackwell redundancy $I_{\cap }^{≺}$ is the maximum information that Bob can have about *Y* without being able to do better than Alice on any utility function, regardless of which source Alice has access to.

Blackwell redundancy can also be used to define a measure of Blackwell unique information, $U^{≺}\left(X_{i}\;\to \;Y|X_{1};\ldots ;X_{n}\right):=I\left(Y;X_{i}\right)-I_{\cap }^{≺}\left(X_{1};\ldots ;X_{n}\;\to \;Y\right)$, via Equation (2). As we show in Appendix I, $U^{≺}$ satisfies the following property, which we term the *Multivariate Blackwell property*.

**Theorem 3.**  *$U^{≺}\left(X_{i}\;\to \;Y|X_{1};\ldots ;X_{n}\right)=0$ if and only if $X_{i}{≺}_{Y}X_{j}$ for all j≠i.*

Operationally, Theorem 3 means that source $X_{i}$ has non-zero unique information iff there exists a utility function such that an agent with access to source $X_{i}$ can achieve higher utility than an agent with access to any other source $X_{j}$.

Computing $I_{\cap }^{≺}$ involves maximizing a convex function subject to a set of linear constraints. These constraints define a feasible set which is a convex polytope, and the maximum must lie on one of the vertices of this polytope [63]. In Appendix C, we show how to solve this optimization problem. In particular, we use a computational geometry package to enumerate the vertices of the feasible set, and then choose the best vertex (code is available at [64]). In that appendix, we also prove that an optimal solution to Equation (27) can always be achieved by *Q* with cardinality $\left|Q\right|=\left(\sum_{i}\left|X_{i}\right|\right)-n+1$. Note that the supremum in Equation (27) is always achieved. Note also that $I_{\cap }^{≺}$ satisfies the redundancy axioms in Section 4.2.

As discussed above, solving the optimization problem in Equation (27) gives a (possibly non-unique) optimal random variable *Q* which specifies the content of the redundant information. As shown in Appendix C, solving Equation (27) also provides a set of channels $\kappa_{Q|X_{i}}$ for each source $X_{i}$, which identify the redundant information in each source.

Note that the Blackwell order satisfies assumptions I-III in Section 4.1, thus Blackwell redundancy satisfies the bounds derived in that section. Finally, note that like many other redundancy measures, Blackwell redundancy becomes equivalent to the measure $I_{\cap }^{\mathrm{MMI}}$ (as defined in Equation (19)) when applied to Gaussian random variables (for details, see Appendix E).

### 5.3. Blackwell Union Information

We now define a measure of union information using our general definition in Equation (8), while using the Blackwell order relative to *Y* as the “more informative” relation:

(28) $$I_{\cup }^{≺}\left(X_{1};\ldots ;X_{n}\;\to \;Y\right):=\underset{Q}{\inf}\;I\left(Q;Y\right)\;\;\mathrm{such}\;\mathrm{that}\;\;\forall i\;X_{i}{≺}_{Y}Q.$$

We refer to this measure as *Blackwell union information*.

As for Blackwell redundancy, Blackwell union information can be understood in operational terms. Consider two agents, Alice and Bob, whose use information about *Y* to maximize their expected utility. Suppose that Alice has access to one of the sources $X_{i}$. Then, the Blackwell union information $I_{\cup }^{≺}$ is the minimum information that Bob must have about *Y* in order to do better than Alice on any utility function, regardless of which source Alice has access to.

Blackwell union information can be used to define measures of synergy and excluded information via Equations (1) and (3). The resulting measure of excluded information $E^{≺}\left(X_{i}\;\to \;Y|X_{1};\ldots ;X_{n}\right):=I_{\cup }^{≺}\left(X_{1};\ldots ;X_{n}\;\to \;Y\right)-I\left(Y;X_{i}\right)$ satisfies the following property, which is the “dual” of the *Multivariate Blackwell property* considered in Theorem 3. (See Appendix I for the proof).

**Theorem 4.**  *$E^{≺}\left(X_{i}\;\to \;Y|X_{1};\ldots ;X_{n}\right)=0$ if and only if $X_{j}{≺}_{Y}X_{i}$ for all j≠i.*

Operationally, Theorem 4 means that there is excluded information for source $X_{i}$ iff there exists a utility function such that an agent with access to one of the other sources $X_{j}$ can achieve higher expected utility than an agent with access to $X_{i}$.

We discuss the problem of numerically solving the optimization problem in Equation (28) in the next subsection.

### 5.4. Relation to Prior Work

Our measure of Blackwell redundancy $I_{\cap }^{≺}$ is new to the PID literature. The most similar existing redundancy measure is $I_{\cap }^{\mathrm{GH}}$ [18], which is discussed above in Section 4.4. $I_{\cap }^{\mathrm{GH}}$ is a special case of Equation (7), once the “more informative” relation B⊏C is defined in terms of conditional independence B−C−Y. Note that conditional independence is stronger than the Blackwell order: given the definition of ${≺}_{Y}$ in Equation (23), it is clear that B−C−Y implies $B{≺}_{Y}C$ (the channel $\kappa_{B|C}$ can be taken to be $P_{B|C}$), but not vice versa. As discussed in Section 4.1, stronger ordering relations give smaller values of redundancy, so in general $I_{\cap }^{\mathrm{GH}}\leq I_{\cap }^{≺}$. Note also that $B{≺}_{Y}C$ depends only on the pairwise marginals $P_{BY}$ and $P_{CY}$, while conditional independence B−C−Y depends on the joint distribution $P_{BCY}$. As we discuss in Appendix F, the conditional independence order can be interpreted in decision-theoretic terms, which suggests an operational interpretation for $I_{\cap }^{\mathrm{GH}}$.

Interestingly, Blackwell union information $I_{\cup }^{≺}$ is equivalent to two measures that have been previously proposed in the PID literature, although they were formulated in a different way. Bertschinger et al. [13] considered the following measure of bivariate redundancy:

(29) $$I_{\cap }^{\mathrm{BROJA}}\left(X_{1};X_{2}\;\to \;Y\right):=I\left(Y;X_{1}\right)+I\left(Y;X_{2}\right)-I_{\cup }^{\mathrm{BROJA}}\left(X_{1};X_{2}\;\to \;Y\right),$$

where $I_{\cup }^{\mathrm{BROJA}}$ is defined via the optimization problem

(30) $$I_{\cup }^{\mathrm{BROJA}}\left(X_{1};X_{2}\;\to \;Y\right)=\min_{{\tilde{X}}_{1},{\tilde{X}}_{2}}I\left(Y;{\tilde{X}}_{1},{\tilde{X}}_{2}\right)\;\;\mathrm{such}\;\mathrm{that}\;\;P_{{\tilde{X}}_{1}Y}=P_{X_{1}Y},P_{{\tilde{X}}_{2}Y}=P_{X_{2}Y},$$

and reflects the minimal mutual information that two random variables can have about *Y*, given that their pairwise marginals with *Y* are fixed to be $P_{X_{1}Y}$ and $P_{X_{2}Y}$. Note that Ref. [13] did not refer to $I_{\cup }^{\mathrm{BROJA}}$ as a measure of union information (we use our notation in writing it as $I_{\cup }^{\mathrm{BROJA}}$). Instead, these measures were derived from an operational motivation, with the goal of deriving a unique information measure that obeys the so-called *Blackwell property*: $I\left(Y;X_{1}\right)-I_{\cap }^{\mathrm{BROJA}}\left(X_{1};X_{2}\;\to \;Y\right)=0$ if $X_{1}{≺}_{Y}X_{2}$ (see Theorems 3 and 4 above).

Starting from a different motivation, Griffith and Koch [17] proposed a multivariate version of $I_{\cup }^{\mathrm{BROJA}}$,

(31) $$I_{\cup }^{\mathrm{BROJA}}\left(X_{1};\ldots ;X_{n}\;\to \;Y\right)=\min_{{\tilde{X}}_{i},\ldots ,{\tilde{X}}_{n}}I\left(Y;{\tilde{X}}_{1},\ldots ,{\tilde{X}}_{n}\right)\;\;\mathrm{such}\;\mathrm{that}\;\;\forall i\;P_{{\tilde{X}}_{i}Y}=P_{X_{i}Y}.$$

The goal of Ref. [17] was to derive a measure of multivariate synergy from a measure of union information, as in Equation (1). In that paper, $I_{\cup }^{\mathrm{BROJA}}$ was explicitly defined as a measure of union information. To our knowledge, Ref. [17] was the first (and perhaps only) paper to propose a measure of union information that was not derived from redundancy via the inclusion-exclusion principle.

While $I_{\cup }^{\mathrm{BROJA}}\left(X_{1};\ldots ;X_{n}\;\to \;Y\right)$ and $I_{\cup }^{≺}\left(X_{1};\ldots ;X_{n}\;\to \;Y\right)$ are stated as different optimization problems, we prove in Appendix G that these optimization problems are equivalent, in that they will always achieve the same optimum value. Interestingly, since $I_{\cup }^{\mathrm{BROJA}}$ and $I_{\cup }^{≺}$ are equivalent, our measure of Blackwell redundancy $I_{\cap }^{≺}$ appears as the natural dual to $I_{\cup }^{\mathrm{BROJA}}$. Another implication of this equivalence is that Blackwell union information $I_{\cup }^{≺}$ can be quantified by solving the optimization problem in Equation (31), rather than Equation (28). This is advantageous, because Equation (31) involves the minimization of a convex function over a convex polytope, which can be solved using standard convex optimization techniques [65].

In Ref. [13], the redundancy measure $I_{\cap }^{\mathrm{BROJA}}$ in Equation (29) was only defined for the bivariate case. Since then, it has been unclear how to extend this redundancy measure to more than two sources. However, by comparing Equations (14) and (29), we see the root of the problem: $I_{\cap }^{\mathrm{BROJA}}$ is derived by applying the inclusion-exclusion principle to a measure of union information, $I_{\cup }^{\mathrm{BROJA}}$. It cannot be extended to more than two sources because the inclusion-exclusion principle generally leads to counterintuitive results for more than 2 sources, as shown in Lemma 1. Note also that what Ref. [13] called the unique information in $X_{1}$, $I_{\cup }^{\mathrm{BROJA}}\left(X_{1};X_{2}\;\to \;Y\right)-I\left(Y;X_{2}\right)$, in our framework would be considered a measure of the excluded information for $X_{2}$.

At the same time, the union information measure $I_{\cup }^{\mathrm{BROJA}}$, and the corresponding synergy from Equation (1), does not use the inclusion-exclusion principle. Therefore, it can be easily extended to any number of sources [17].

### 5.5. Continuity of Blackwell Redundancy and Union Information

It is often desired that information-theoretic measures are continuous, meaning that small changes in underlying probability distributions lead to small changes in the resulting measures. In this section, we consider the continuity of our proposed measures, $I_{\cap }^{≺}$ and $I_{\cup }^{≺}$.

We first consider Blackwell redundancy $I_{\cap }^{≺}$. It turns out that this measure is not always continuous in the joint probability $P_{X_{1}\ldots X_{n}Y}$ (a discontinuous example is provided in Section 5.6). However, the discontinuity of $I_{\cap }^{≺}$ is not necessarily pathological, and we can derive an interpretable geometric condition that guarantees that $I_{\cap }^{≺}$ is continuous.

Consider the conditional distribution of the target *Y* given some source $X_{i}$, $P_{Y|X_{i}}$. Let $\mathrm{rank}\;P_{Y|X_{i}}$ indicate its *rank*, meaning the dimension of the space spanned by the vectors ${\left\{P_{Y|X_{i}=x_{i}}\right\}}_{x_{i}\in X_{i}}$. The rank of $P_{Y|X_{i}}$ quantifies the number of independent directions that the target distribution $P_{Y}$ can be moved by manipulating the source distribution $P_{X_{i}}$, and it cannot be larger than |Y|. The next theorem shows that $I_{\cap }^{≺}$ is locally continuous, as long as n−1 or more of the source conditional distributions have this maximal rank.

**Theorem 5.**  *As a function of the joint distribution $P_{X_{1},\ldots ,X_{n},Y}$, $I_{\cap }^{≺}$ is locally continuous whenever n−1 or more of the conditional distributions $P_{Y|X_{i}}$ have $\mathrm{rank}\;P_{Y|X_{i}}=\left|Y\right|$.*

In proving this result, we also show that $I_{\cap }^{≺}$ is continuous almost everywhere (see proof in Appendix D). Finally, in that appendix we also use Theorem 5 to show that $I_{\cap }^{≺}$ is continuous everywhere if *Y* is a binary random variable.

**Figure 2:**
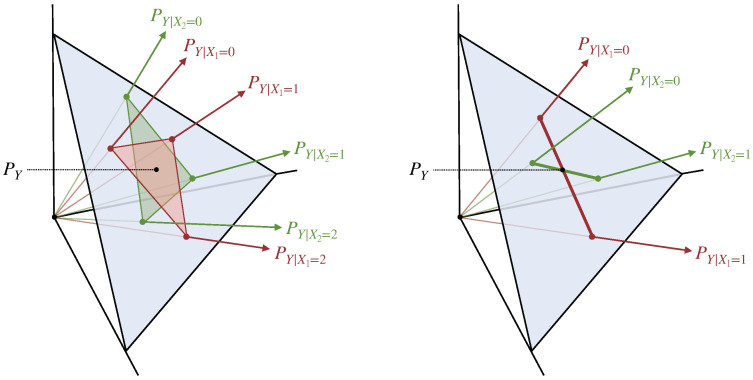
Illustration of Theorem 5, which provides a sufficient condition for the local continuity of $I_{\cap }^{≺}$. Consider two scenarios, both of which involves two sources $X_{1}$ and $X_{2}$ and a target *Y* with cardinality |Y|=3. The blue areas indicate the simplex of probability distributions over Y, with the marginal $P_{Y}$ and the pairwise conditionals $P_{Y|X_{i}=x_{i}}$ marked. On the left, both sources have $\mathrm{rank}\;P_{Y|X_{i}}=3=\left|Y\right|$, so $I_{\cap }^{≺}$ is locally continuous. On the right, both sources have $\mathrm{rank}\;P_{Y|X_{i}}=2<\left|Y\right|$, so $I_{\cap }^{≺}$ is not necessarily locally continuous. Note that $I_{\cap }^{≺}$ is also continuous if only source has $\mathrm{rank}\;P_{Y|X_{i}}=3$.

We illustrate the meaning of Theorem 5 visually in Figure 2. We show two situations, both of which involve two sources $X_{1}$ and $X_{2}$ and a target *Y* with cardinality |Y|=3. In one situation, both pairwise conditional distributions have rank equal to |Y|, so $I_{\cap }^{≺}$ is locally continuous. In the other situation, both pairwise conditional distributions are rank deficient (e.g., this might happen because $X_{1}$ and $X_{2}$ have cardinality $|X_{1}\left|=\right|X_{2}|=2$), so $I_{\cap }^{≺}$ is not guaranteed to be continuous. From the figure it is easy to see how the discontinuity may arise. Given the definition of the Blackwell order and $I_{\cap }^{≺}$, for any random variable *Q* in the feasible set of Equation (27), the conditional distributions $P_{Y|Q=q}$ must fall within the intersection of the distributions spanned by $P_{Y|X_{1}}$ and $P_{Y|X_{2}}$ (the intersection of the red and green shaded regions in Figure 2). On the right, the size of this intersection can discontinuously jump from a line (when $P_{Y|X_{1}}$ and $P_{Y|X_{2}}$ are perfectly aligned) to a point (when $P_{Y|X_{1}}$ and $P_{Y|X_{2}}$ are not perfectly aligned). Thus, the discontinuity of $I_{\cap }^{≺}$ arises from a geometric phenomenon, which is related to the discontinuity of the intersection of low-dimensional vector subspaces.

We briefly comment on the continuity of $I_{\cup }^{≺}$. As we described above, this measure turns out to be equivalent to $I_{\cup }^{\mathrm{BROJA}}$. The continuity of $I_{\cup }^{\mathrm{BROJA}}$ in the bivariate case was proven in Theorem 35 of Ref. [66]. We believe that the continuity of $I_{\cup }^{\mathrm{BROJA}}$ for an arbitrary number of sources can be shown using similar methods, although we leave this for future work.

### 5.6. Behavior on the COPY Gate

As mentioned in Section 3, the “COPY gate” example is often used to test the behavior of different redundancy measures. The COPY gate has two sources, $X_{1}$ and $X_{2}$, and a target $Y=(X_{1},X_{2})$ which is a copy of the joint outcome. It is expected that redundancy should vanish if $X_{1}$ and $X_{2}$ are statistically independent, as formalized by the *Independent identity* property in Equation (4).

Blackwell redundancy $I_{\cap }^{≺}$ satisfies the *Independent identity*. In fact, we prove a more general result, which shows that $I_{\cap }^{≺}\left(X_{1},X_{2}\;\to \;\left(X_{1},X_{2}\right)\right)$ is equal to an information-theoretic measure called *Gács-Körner common information* C(X∧Y) [16,47,67]. C(X∧Y) quantifies the amount of information that can be deterministically extracted from both random variables *X* or *Y*, and it is closely related to the “deterministic function” order ⊲ defined in Equation (16). Formally, it can be written as

(32) $$C\left(X\wedge Y\right)=\underset{Q}{\sup}H\left(Q\right)\;\;\mathrm{such}\;\mathrm{that}\;\;Q⊲X,Q⊲Y,$$

where *H* is Shannon entropy. In Appendix I, we prove the following result.

**Theorem 6.**  *$I_{\cap }^{≺}\left(X_{1},X_{2}\;\to \;\left(X_{1},X_{2}\right)\right)=C\left(X_{1}\wedge X_{2}\right)$.*

Note that $0\leq C\left(X_{1}\wedge X_{2}\right)\leq I\left(X_{1};X_{2}\right)$ [47], so $I_{\cap }^{≺}$ satisfies the *Independent identity* property. At the same time, $C(X_{1}\wedge X_{2})$ can be strictly less than $I(X_{1};X_{2})$. For example, if $P_{X_{1}X_{2}}$ has full support, then $I(X_{1};X_{2})$ can be arbitrarily large while $C(X_{1}\wedge X_{2})=0$ (see proof of Theorem 6). This means that $I_{\cap }^{≺}$ violates a previously proposed property, sometimes called the *Identity property*, that suggests that redundancy should satisfy $I_{\cap }\left(X_{1};X_{2}\;\to \;\left(X_{1},X_{2}\right)\right)=I\left(X_{1};X_{2}\right)$. However, the validity of the *Identity property* is not clear, and several papers have argued against it [15,39].

The value of $C(X_{1}\wedge X_{2})$ depends on the precise pattern of zeros in the joint distribution $P_{X_{1}X_{2}}$ and is therefore not continuous. For instance, for the bivariate COPY gate, redundancy can change discontinuously as one goes from the situation where $X_{1}=X_{2}$ (so that all information is redundant, $I_{\cap }^{≺}=I\left(X_{1};X_{2}\right)$) to one where $X_{1}$ and $X_{2}$ are almost, but not entirely, identical. This discontinuity can be understood in terms of Theorem 5 and Figure 2: in the COPY gate, the cardinality of the target variable $\left|Y|=\right|X_{1}\left|\times \right|X_{2}|$ is larger than the cardinality of the individual sources. In other words, when the sources $X_{1}$ and $X_{2}$ are not perfectly correlated, they provide information about different “subspaces” of the target $\left(X_{1},X_{2}\right)$, and so it is possible that very little (or none) of their information is redundant.

At the same time, the Blackwell property, Theorem 3, implies that

(33) $$I_{\cap }^{≺}\left(X_{1},X_{2}\;\to \;X_{1}\right)=I\left(X_{1};X_{2}\right)=I_{\cap }^{≺}\left(X_{1},X_{2}\;\to \;X_{2}\right)$$

In other words, the redundancy in $X_{1}$ and $X_{2}$, where either one of the individual sources is taken as the target, is given by the mutual information $I(X_{1};X_{2})$. This holds even though the redundancy in the COPY gate can be much lower than $I(X_{1};X_{2})$.

It is also interesting to consider how Blackwell union information, $I_{\cup }^{≺}$, behaves on the COPY gate. Using techniques from [13], it can be shown that the union information is simply the joint entropy,

(34) $$\begin{array}{cc} I_{\cup }^{≺}\left(X_{1};X_{2}\;\to \;\left(X_{1},X_{2}\right)\right) & =H(X_{1},X_{2}). \end{array}$$

Since $H\left(X_{1},X_{2}\right)=I\left(X_{1},X_{2};X_{1},X_{2}\right)$, Equations (1) and (34) together imply that the COPY gate has no synergy.

Note that we can use Theorem 6 and Equation (34) to illustrate that $I_{\cap }^{≺}$ and $I_{\cup }^{≺}$ violate the inclusion-exclusion principle, Equation (14). Using Equation (34) and a bit of rearranging, Equation (14) becomes equivalent to $I_{\cap }^{≺}\left(X_{1};X_{2}\;\to \;\left(X_{1},X_{2}\right)\right)\overset{?}{=}I\left(X_{1};X_{2}\right)$, which is the *Identity property* mentioned above. $I_{\cap }^{≺}$ violates this property, since redundancy for the COPY gate can be smaller than $I(X_{1};X_{2})$.

## 6. Examples and Comparisons to Previous Measures

In this section, we compare our proposed measure of Blackwell redundancy $I_{\cap }^{≺}$ to existing redundancy measures. We focus on redundancy, rather than union information, because redundancy has seen much more development in the literature, and because Blackwell union information $I_{\cup }^{≺}$ is equivalent to an existing measure (see Section 5.4).

### 6.1. Qualitative Comparison

In Table 1, we compare $I_{\cap }^{≺}$ to six existing measures of multivariate redundancy:

- $I_{\cap }^{\mathrm{WB}}$, the redundancy measure first proposed by Williams and Beer [11].
- $I_{\cap }^{\mathrm{MMI}}$, the “minimum mutual information” [51], Equation (19) in Section 4.4.
- $I_{\cap }^{⊲}$, proposed by Griffith et al. [16], Equation (17) in Section 4.4.
- $I_{\cap }^{\mathrm{GH}}$, proposed by Griffith and Ho [18], Equation (18) in Section 4.4.
- $I_{\cap }^{\mathrm{Ince}}$, proposed by Ince [20].
- $I_{\cap }^{\mathrm{FL}}$, proposed by Finn and Lizier [21].

We also compare $I_{\cap }^{≺}$ to three existing measures of bivariate redundancy (i.e., for 2 sources):

- $I_{\cap }^{\mathrm{BROJA}}$, proposed by Bertschinger et al. [13], defined in Equation (29).
- $I_{\cap }^{\mathrm{Harder}}$, proposed by Harder et al. [19].
- $I_{\cap }^{\mathrm{dep}}$, proposed by James et al. [15].

For $I_{\cap }^{≺}$ as well as the 9 existing measures, we consider the following properties, which are chosen to highlight differences between our approach and previous proposals:

1. Has it been defined for more than 2 sources
2. Does it obey the *Monotonicity* axiom from Section 4.2
3. Is it compatible with the inclusion-exclusion principle (IEP) for the bivariate case, such that union information as defined in Equation (14) obeys $I_{\cup }\left(X_{1};X_{2}\;\to \;Y\right)\leq I\left(X_{1},X_{2};Y\right)$
4. Does it obey the *Independent identity* property, Equation (4)
5. Does it obey the *Blackwell property* (possibly in its multivariate form, Theorem 3)

We also consider two additional properties, which require a bit of introduction.

The first property was suggested by Ref. [13], who argued that redundancy should only depend on the pairwise marginal distributions of each source with the target,

(35) $$\mathrm{If}\;p_{X_{i}Y}=p_{{\tilde{X}}_{i}\tilde{Y}}\;\mathrm{for}\;\mathrm{all}\;i,\;\mathrm{then}\;I_{\cap }\left(X_{1};\ldots ;X_{n}\;\to \;Y\right)=I_{\cap }\left({\tilde{X}}_{1};\ldots ;{\tilde{X}}_{n}\;\to \;\tilde{Y}\right).$$

In Table 1, we term this property *Pairwise marginals.* We believe that the validity of Equation (35) is not universal, but may depend on the particular setting in which the PID is being used. However, redundancy redundancy measures that satisfy this property have one important advantage: they are well-defined not only when the sources are random variables $X_{1},\ldots ,X_{n}$, but also in the more general case when the sources are channels $\kappa_{X_{1}|Y},\ldots ,\kappa_{X_{n}|Y}$.

The second property has not been previously considered in the literature, although it appears to be highly intuitive. Observe that the target random variable *Y* contains all possible information about itself. Thus, it may be expected that adding the target to the set of sources should not decrease the redundancy:

(36) $$I_{\cap }\left(X_{1};\ldots ;X_{n};Y\;\to \;Y\right)=I_{\cap }\left(X_{1};\ldots ;X_{n}\;\to \;Y\right).$$

In Table 1, we term this property *Target equality*. Note that for redundancy measures which can be put in the form of Equation (7), *Target Equality* is satisfied if the order ⊏ obeys $X_{i}⊏Y$ for all sources $X_{i}$. (Note also that *Target Equality* is unrelated to the previously proposed *Strong Symmetry* property; for instance, it is easy to show that the redundancy measures $I_{\cap }^{\mathrm{WB}}$ and $I_{\cap }^{\mathrm{MMI}}$ satisfy *Target Equality*, even though they violate *Strong Symmetry* [68]).

### 6.2. Quantitative Comparison

We now illustrate our proposed measure of redundancy $I_{\cap }^{≺}$ on some simple examples, and compare its behavior to existing redundancy measures.

The values of $I_{\cap }^{≺}$ were computed with our code, provided at [64]. The values of all other redundancy measures except $I_{\cap }^{\mathrm{GH}}$ were computed using the dit Python package [69]. To our knowledge, there have been no previous proposals for how to compute $I_{\cap }^{\mathrm{GH}}$. In fact, this measure involves maximizing a convex function subject to linear constraints, and can be computed using similar methods as $I_{\cap }^{≺}$. We provide code for computing $I_{\cap }^{\mathrm{GH}}$ at [64].

We begin by considering some simple bivariate examples. In all cases, the sources $X_{1}$ and $X_{2}$ are binary and uniformly distributed. The results are shown in Table 2.

1. The AND gate, $Y=X_{1}\;\mathrm{AND}\;X_{2}$, with $X_{1}$ and $X_{2}$ independent. (It is incorrectly stated in Refs. [18,49] that $I_{\cap }^{\mathrm{GH}}$ vanishes here; actually $I_{\cap }^{\mathrm{GH}}\left(X_{1};X_{2}\;\to \;X_{1}\;\mathrm{AND}\;X_{2}\right)\approx 0.123$, which corresponds to the maximum achieved in Equation (18) by $Q=X_{1}\;\mathrm{OR}\;X_{2}$.)
2. The SUM gate: $Y=X_{1}+X_{2}$, with $X_{1}$ and $X_{2}$ independent.
3. The UNQ gate: $Y=X_{1}$. Here $I_{\cap }^{\mathrm{Ince}}$ (marked with ∗) gave values that increased with the amount of correlation between $X_{1}$ and $X_{2}$ but were typically larger than $I(X_{1};\;X_{2})$.
4. The COPY gate: $Y=(X_{1},X_{2})$. Here, our redundancy measure is equal to the Gács-Körner common information between *X* and *Y*, as discussed in Section 5.6. The same holds for the redundancy measures $I_{\cap }^{\mathrm{GH}}$ and $I_{\cap }^{⊲}$, which can be shown using a slight modification of the proof of Theorem 6. For this gate, $I_{\cap }^{\mathrm{Ince}}$ (marked with ∗) gave the same values as for the UNQ gate, which increased with the amount of correlation between $X_{1}$ and $X_{2}$ but were typically larger than $I(X_{1};X_{2})$.

We also analyze several examples with three sources, with the results shown in Table 3. We considered those previously proposed measures which can be applied to more than two sources (we do not show $I_{\cap }^{\mathrm{GH}}$, as our implementation was too slow for these examples).

1. Three-way AND gate: $Y=X_{1}\mathrm{AND}X_{2}\mathrm{AND}X_{3}$, where the sources are binary and uniformly and independently distributed.
2. Three-way SUM gate: $Y=X_{1}+X_{2}+X_{3}$, where the sources are binary and uniformly and independently distributed.
3. “Overlap” gate: we defined four independent uniformly distributed binary random variables, A,B,C,D. These were grouped into three sources $X_{1},X_{2},X_{3}$ as $X_{1}=\left(A,B\right)$, $X_{2}=\left(A,C\right)$, $X_{3}=\left(A,D\right)$. The target was the joint outcome of all three sources, $Y=\left(X_{1},X_{2},X_{3}\right)=\left(\left(A,B\right),\left(A,C\right),\left(A,D\right)\right)$. Note that the three sources overlap on a single random variable *A*, which suggests that the redundancy should be 1 bit.

## 7. Discussion and Future Work

In this paper, we proposed a new general framework for defining the partial information decomposition (PID). Our framework was motivated in several ways, including a formal analogy with intersections and unions in set theory as well as an axiomatic derivation.

We also used our general framework to propose concrete measures of redundancy and union information, which have clear operational interpretations based on Blackwell’s theorem. Other PID measures, such as synergy and unique information, can be computed from our measures of redundancy and union information via simple expressions.

One unusual aspect of our framework is that it provides separate measures of redundancy and union information. As we discuss above, most prior work on the PID assumed that redundancy and union information are related to each other via the so-called “inclusion-exclusion” principle. We argue that the inclusion-exclusion principle should not be expected to hold in the context of the PID, and in fact that it leads to counterintuitive behavior once 3 or more sources are present. This suggests that different information decompositions should be derived for redundancy vs. union information. This idea is related to a recent proposal in the literature, which argues that two different PIDs are needed, one based on redundancy and one based on synergy [41]. An interesting direction for future work is to relate our framework with the dual decompositions proposed in [41].

From a practical standpoint, an important direction for future work is to develop better schemes for computing our redundancy measure. This measure is defined in terms of a convex maximization problem, which in principle can be NP-hard (a similar convex maximization problem was proven to be NP-hard in [70]). Our current implementation, which enumerates the vertices of the feasible set, works well for relatively small state spaces, but we do not expect it to scale to situations with many sources, or where the sources have large cardinalities. However, the problem of convex maximization with linear constraints is a very active area of optimization research, with many proposed algorithms [63,71,72]. Investigating these algorithms, as well as various approximation schemes such as relaxations and variational bounds, is of interest.

Finally, we showed how our framework can be used to define measures of redundancy and union information in situations that go beyond the standard setting of the PID (e.g., when the probability distribution of the target is not specified). Our framework can even be applied in domains beyond Shannon information theory, such as algorithmic information theory and quantum information theory. Future work may exploit this flexibility to explore various new applications of the PID. 

## Figures and Tables

**Table 1:** Comparison of different redundancy measures. ? indicate properties that we could not easily establish.

	$I_{\cap }^{≺}$	$I_{\cap }^{\mathrm{WB}}$	$I_{\cap }^{\mathrm{MMI}}$	$I_{\cap }^{⊲}$	$I_{\cap }^{\mathrm{GH}}$	$I_{\cap }^{\mathrm{Ince}}$	$I_{\cap }^{\mathrm{FL}}$	$I_{\cap }^{\mathrm{BROJA}}$	$I_{\cap }^{\mathrm{Harder}}$	$I_{\cap }^{\mathrm{dep}}$
More than 2 sources	✓	✓	✓	✓	✓	✓	✓			
Monotonicity	✓	✓	✓	✓	✓			✓	✓	✓
IEP for bivariate case		✓	✓			?	?	✓	✓	✓
Independent identity	✓			✓	✓	✓		✓	✓	✓
Blackwell property	✓							✓	✓	
Pairwise marginals	✓	✓	✓	✓				✓	✓	
Target equality	✓	✓	✓		✓			✓	✓	

**Table 2:** Behavior of $I_{\cap }^{≺}$ and other redundancy measures on bivariate examples.

Target	$I_{\cap }^{≺}$	$I_{\cap }^{\mathrm{WB}}$	$I_{\cap }^{\mathrm{MMI}}$	$I_{\cap }^{\wedge }$	$I_{\cap }^{\mathrm{GH}}$	$I_{\cap }^{\mathrm{Ince}}$	$I_{\cap }^{\mathrm{FL}}$	$\begin{array}{c} I_{\cap }^{\mathrm{BROJA}}\\ I_{\cap }^{\mathrm{Harder}} \end{array}$	$I_{\cap }^{\mathrm{dep}}$
$Y=X_{1}\;\mathrm{AND}\;X_{2}$	0.311	0.311	0.311	0	0.123	0.104	0.561	0.311	0.082
$Y=X_{1}+X_{2}$	0.5	0.5	0.5	0	0	0	0.5	0.5	0.189
$Y=X_{1}$	$I(X_{1};\;X_{2})$	$I(X_{1};\;X_{2})$	$I(X_{1};\;X_{2})$	$C(X_{1}\;\wedge \;X_{2})$	$I(X_{1};\;X_{2})$	*	1	$I(X_{1};\;X_{2})$	$I(X_{1};\;X_{2})$
$Y=(X_{1},X_{2})$	$C(X_{1}\;\wedge \;X_{2})$	1	1	$C(X_{1}\;\wedge \;X_{2})$	$C(X_{1}\;\wedge \;X_{2})$	*	1	$I(X_{1};\;X_{2})$	$I(X_{1};\;X_{2})$

**Table 3:** Behavior of $I_{\cap }^{≺}$ and other redundancy measures on three sources.

Target	$I_{\cap }^{≺}$	$I_{\cap }^{\mathrm{WB}}$	$I_{\cap }^{\mathrm{MMI}}$	$I_{\cap }^{\wedge }$	$I_{\cap }^{\mathrm{Ince}}$	$I_{\cap }^{\mathrm{FL}}$
$Y=X_{1}\;\mathrm{AND}\;X_{2}\;\mathrm{AND}\;X_{3}$	0.138	0.138	0.138	0	0.024	0.294
$Y=X_{1}+X_{2}+X_{3}$	0.311	0.311	0.311	0	0	0.561
Y=((A,B),(A,C),(A,D))	1	2	2	1	1	2

## Data Availability

Not applicable.

## Appendix A. PID Axioms

In developing the PID framework, Williams and Beer [11,12] proposed that any measure of redundancy should obey a set of axioms. In slightly modified form, these axioms can be written as follows:

- *Symmetry*: $I_{\cap }\left(X_{1};\ldots ;X_{n}\;\to \;Y\right)$ is invariant to the permutation of $X_{1},\ldots ,X_{n}$.
- *Self-redundancy*: $I_{\cap }\left(X_{1}\;\to \;Y\right)=I\left(Y;X_{1}\right)$.
- *Monotonicity*: $I_{\cap }\left(X_{1};\ldots ;X_{n}\;\to \;Y\right)\leq I_{\cap }\left(X_{1};\ldots ;X_{n-1}\;\to \;Y\right)$.
- *Deterministic equality:*$I_{\cap }\left(X_{1};\ldots ;X_{n}\;\to \;Y\right)=I_{\cap }\left(X_{1};\ldots ;X_{n-1}\;\to \;Y\right)$ if $X_{i}=f\left(X_{n}\right)$ for some i<n and deterministic function *f*.

These axioms are based on intuitions regarding the behavior of intersection in set theory [12]. The *Symmetry* axiom is self-explanatory. *Self-redundancy* states that if only a single-source is present, all of its information is redundant. *Monotonicity* states that redundancy should not increase when an additional source is considered (consider that the size of set intersection can only decrease as more sets are considered). *Deterministic equality* states that redundancy should remain the same when an additional source $X_{n}$ is added that contains all (or more) of the same information that is already contained in an existing source $X_{i}$ (which is formalized as the condition $X_{i}=f\left(X_{n}\right)$).

Union information was considered the original PID proposal [12,73], as well as a more recent paper [17]. Ref. [17] proposed that any measure of union information should satisfy the following set of natural axioms, stated here in slightly modified form:

- *Symmetry*: $I_{\cup }\left(X_{1};\ldots ;X_{n}\;\to \;Y\right)$ is invariant to the permutation of $X_{1},\ldots ,X_{n}$.
- *Self-union*: $I_{\cup }\left(X_{1}\;\to \;Y\right)=I\left(Y;X_{1}\right)$.
- *Monotonicity*: $I_{\cup }\left(X_{1};\ldots ;X_{n}\;\to \;Y\right)\geq I_{\cup }\left(X_{1};\ldots ;X_{n-1}\;\to \;Y\right)$.
- *Deterministic equality*: $I_{\cup }\left(X_{1};\ldots ;X_{n}\;\to \;Y\right)=I_{\cup }\left(X_{1};\ldots ;X_{n-1}\;\to \;Y\right)$ if $X_{n}=f\left(X_{i}\right)$ for some i<n and deterministic function *f*.

These axioms are based on intuitions concerning the behavior of the union operator in set theory, and are the natural “duals” of the redundancy axioms mentioned above.

## Appendix B. Uniqueness Proofs

**Proof of Theorem 1.** Assume there is a redundancy measure $I_{\cap }^{\prime }$ that obeys the five axioms stated in the theorem. We will show that $I_{\cap }^{\prime }=I_{\cap }$, as defined in Equation (7). Given Equation (7) and the definition of the supremum, for any ϵ>0 there exists a random variable *Q* such that $Q⊏X_{i}$ for i∈{1,…,n} and

(A1) $$I\left(Q;Y\right)\geq I_{\cap }\left(X_{1};\ldots ;X_{n}\;\to \;Y\right)-\epsilon ,$$

By *Order equality*, $I_{\cap }^{\prime }\left(Q;X_{1};\ldots ;X_{k}\;\to \;Y\right)=I_{\cap }^{\prime }\left(Q;X_{1};\ldots ;X_{k-1}\;\to \;Y\right)$. Induction gives

$$I_{\cap }^{\prime }\left(Q;X_{1};\ldots ;X_{n}\;\to \;Y\right)=I_{\cap }^{\prime }\left(Q\;\to \;Y\right)=I\left(Q;Y\right)\geq I_{\cap }\left(X_{1};\ldots ;X_{n}\;\to \;Y\right)-\epsilon$$

where we used *Self-redundancy* and Equation (A1). We also have $I_{\cap }^{\prime }\left(Q;X_{1};\ldots ;X_{n}\;\to \;Y\right)\leq I_{\cap }^{\prime }\left(X_{1};\ldots ;X_{n}\;\to \;Y\right)$ by *Symmetry* and *Monotonicity*. Combining gives

$$I_{\cap }\left(X_{1};\ldots ;X_{n}\;\to \;Y\right)-\epsilon \leq I_{\cap }^{\prime }\left(X_{1};\ldots ;X_{n}\;\to \;Y\right).$$

We now show that $I_{\cap }$ is the largest measure that satisfies *Existence*. Let *Q* be a random variable that obeys $Q⊏X_{i}$ for all i∈{1,…,n} and $I_{\cap }^{\prime }\left(X_{1};\ldots ;X_{n}\;\to \;Y\right)=I\left(Y;Q\right)$. Since *Q* falls within the feasible set of the optimization problem in Equation (7),

$$I_{\cap }^{\prime }\left(X_{1};\ldots ;X_{n}\;\to \;Y\right)=I\left(Q;Y\right)\leq I_{\cap }\left(X_{1};\ldots ;X_{n}\;\to \;Y\right).$$

Combining gives

$$I_{\cap }^{\prime }\left(X_{1};\ldots ;X_{n}\;\to \;Y\right)-\epsilon \leq I_{\cap }\left(X_{1};\ldots ;X_{n}\;\to \;Y\right)-\epsilon \leq I_{\cap }^{\prime }\left(X_{1};\ldots ;X_{n}\;\to \;Y\right).$$

Since this holds for all ϵ>0, taking the limit ϵ→0 gives $I_{\cap }^{\prime }=I_{\cap }$. □

**Proof of Theorem 2.** Assume there is a union information measure $I_{\cup }^{\prime }$ that obeys the five axioms stated in the theorem. We will show that $I_{\cup }^{\prime }=I_{\cup }$, as defined in Equation (8). Given Equation (8) and the definition of the infinitum, for any ϵ>0 there exists a random variable *Q* such that $Q⊏X_{i}$ for i∈{1,…,n} and

(A2) $$I\left(Q;Y\right)\leq I_{\cup }\left(X_{1};\ldots ;X_{n}\;\to \;Y\right)+\epsilon ,$$

By *Order equality*, $I_{\cup }^{\prime }\left(Q;X_{1};\ldots ;X_{k}\;\to \;Y\right)=I_{\cup }^{\prime }\left(Q;X_{1};\ldots ;X_{k-1}\;\to \;Y\right)$. Induction gives

$$I_{\cup }^{\prime }\left(Q;X_{1};\ldots ;X_{n}\;\to \;Y\right)=I_{\cup }^{\prime }\left(Q\;\to \;Y\right)=I\left(Q;Y\right)\leq I_{\cup }\left(X_{1};\ldots ;X_{n}\;\to \;Y\right)+\epsilon$$

where we used *Self-union* and Equation (A2). We also have $I_{\cup }^{\prime }\left(Q;X_{1};\ldots ;X_{n}\;\to \;Y\right)\geq I_{\cup }^{\prime }\left(X_{1};\ldots ;X_{n}\;\to \;Y\right)$ by *Symmetry* and *Monotonicity*. Combining gives

$$I_{\cup }\left(X_{1};\ldots ;X_{n}\;\to \;Y\right)+\epsilon \geq I_{\cup }^{\prime }\left(X_{1};\ldots ;X_{n}\;\to \;Y\right).$$

We now show that $I_{\cap }$ is the smallest measure that satisfies *Existence*. Let *Q* be a random variable that obeys $X_{i}⊏Q$ for all i∈{1,…,n} and $I_{\cup }^{\prime }\left(X_{1};\ldots ;X_{n}\;\to \;Y\right)=I\left(Y;Q\right)$. Since *Q* falls within the feasible set of the optimization problem in Equation (8),

$$I_{\cup }^{\prime }\left(X_{1};\ldots ;X_{n}\;\to \;Y\right)=I\left(Q;Y\right)\geq I_{\cup }\left(X_{1};\ldots ;X_{n}\;\to \;Y\right).$$

Combining gives

$$I_{\cup }^{\prime }\left(X_{1};\ldots ;X_{n}\;\to \;Y\right)\leq I_{\cup }\left(X_{1};\ldots ;X_{n}\;\to \;Y\right)+\epsilon \leq I_{\cap }^{\prime }\left(X_{1};\ldots ;X_{n}\;\to \;Y\right)+\epsilon .$$

Since this holds for all ϵ>0, taking the limit ϵ→0 gives $I_{\cup }^{\prime }=I_{\cup }$. □

## Appendix C. Computing $I_{\cap }^{≺}$

Here we consider the optimization problem that defines our proposed measure of redundancy, Equation (27). We first prove a bound on the required cardinality of *Q*.

**Theorem A1.** 
*For optimizing Equation *(27)*, it suffices to consider Q with cardinality $\left|Q\right|=\left(\sum_{i}\left|X_{i}\right|\right)-n+1$.*

**Proof.** Consider any random variable *Q* with outcome set Q which satisfies $Q{≺}_{Y}X_{i}$ for all *i*. We show that whenever *Q* has full support on $\left|Q\right|>\left(\sum_{i}\left|X_{i}\right|\right)-n+1$ outcomes, there is another random variable $\tilde{Q}$ which achieves $I\left(\tilde{Q};Y\right)\geq I\left(Q;Y\right)$, while satisfying $\tilde{Q}{≺}_{Y}X_{i}$ for all *i* and having support on at most $\left(\sum_{i}\left|X_{i}\right|\right)-n+1$ outcomes. To begin, let Ω indicate the set of random variables over outcomes Q, such that all $\tilde{Q}\in \Omega$ satisfy:

(A3) $$\begin{array}{cccc} P_{Y|\tilde{Q}}\left(y|q\right) & =P_{Y|Q}\left(y|q\right) & \; & \mathrm{for}\;\mathrm{all}\;y,q\in \{q\in Q:P_{\tilde{Q}}\left(q\right)>0\} \end{array}$$

(A4) $$\begin{array}{cccc} \sum_{q}P_{\tilde{Q}}\left(q\right)P_{X_{i}|Q}\left(x_{i}|q\right) & =P_{X_{i}}\left(x_{i}\right)\;\; & \; & \mathrm{for}\;\mathrm{all}\;i,x_{i}. \end{array}$$

Since $Q{≺}_{Y}X_{i}$, by Equation (23) there exist channels $\kappa_{Q|X_{i}}\left(q|x_{i}\right)$ that satisfy $P_{Q|Y}\left(q|y\right)=\sum_{x_{i}}\kappa_{Q|X_{i}}\left(q|x_{i}\right)P_{X_{i}|Y}\left(x_{i}|y\right).$ Now write the conditional distribution over $\tilde{Q}$ and *Y* as

(A5) $$\begin{array}{cc} P_{\tilde{Q}Y}\left(q,y\right)=\frac{P_{\tilde{Q}}\left(q\right)}{P_{Q}\left(q\right)}P_{QY}\left(q,y\right) & =\sum_{x_{i}}\frac{P_{\tilde{Q}}\left(q\right)}{P_{Q}\left(q\right)}\kappa_{Q|X_{i}}\left(q|x_{i}\right)P_{X_{i}|Y}\left(x_{i}|y\right)P_{Y}\left(y\right)\\ & =\sum_{x_{i}}\kappa_{\tilde{Q}|X_{i}}^{\prime }\left(q|x_{i}\right)P_{X_{i}|Y}\left(x_{i}|y\right)P_{Y}\left(y\right), \end{array}$$

where we used Equation (A3) and defined the channel $\kappa_{\tilde{Q}|X_{i}}^{\prime }$ as

$$\kappa_{\tilde{Q}|X_{i}}^{\prime }=\frac{P_{\tilde{Q}}\left(q\right)}{P_{X_{i}}\left(x_{i}\right)}\left[\frac{P_{X_{i}}\left(x_{i}\right)}{P_{Q}\left(q\right)}\kappa_{Q|X_{i}}\left(q|x_{i}\right)\right],$$

(Note this is a kind of double Bayesian inverse, given Equation (A4)). Equation (A5) implies that $\tilde{Q}{≺}_{Y}X_{i}$ for all *i*. We now show that there is $\tilde{Q}\in \Omega$ that achieves $I\left(Q;Y\right)\leq I\left(\tilde{Q};Y\right)$ and has support on at most $\left(\sum_{i}\left|X_{i}\right|\right)-n+1$ outcomes in Q. Write the mutual information between any $\tilde{Q}\in \Omega$ and *Y* as

(A6) $$I\left(\tilde{Q};Y\right)=\sum_{q}P_{\tilde{Q}}\left(q\right)D_{\mathrm{KL}}\left(P_{Y|\tilde{Q}=q}∥P_{Y}\right)=\sum_{q}P_{\tilde{Q}}\left(q\right)D_{\mathrm{KL}}\left(P_{Y|Q=q}∥P_{Y}\right),$$

where $D_{\mathrm{KL}}$ is the Kullback-Leibler divergence. We consider the maximum of this mutual information across Ω, $I^{*}=\max_{\tilde{Q}\in \Omega }I\left(\tilde{Q};Y\right)$. Using Equations (A4) and (A6), this maximum can be written as

$$\begin{array}{cc} I^{*} & =\max_{\omega \in \Delta }\sum_{q}\omega \left(q\right)D\left(P_{Y|Q=q}∥P_{Y}\right)\;\;\mathrm{such}\;\mathrm{that}\;\;\forall i,x_{i}:\sum_{q}\omega \left(q\right)P_{X_{i}|Q}\left(x_{i}|q\right)=P_{X_{i}}\left(x_{i}\right), \end{array}$$

where Δ is the set of all distributions over Q. By conservation of probability, $\sum_{x_{i}}P_{X_{i}}\left(x_{i}\right)=1$, so we can eliminate a constraint for one of the outcomes $x_{i}$ of each source *i*. Thus, $I^{*}$ is the maximum of a linear function over Δ, subject to $\sum_{i}\left(\left|X_{i}\right|-1\right)=\left(\sum_{i}\left|X_{i}\right|\right)-n$ hyperplane constraints. The feasible set is compact, and the maximum will be achieved at one of the extreme points of the feasible set. By Dubin’s Theorem [74], any extreme point of this feasible set can be expressed as a convex combination of at most $\left(\sum_{i}\left|X_{i}\right|\right)-n+1$ extreme points of Δ. In other words, the maximum in Equation (27) is achieved by a random variable $\tilde{Q}$ with support on at most $\left(\sum_{i}\left|X_{i}\right|\right)-n+1$ values of Q. This random variable satisfies

$$I\left(\tilde{Q};Y\right)=I^{*}\geq I\left(Q;Y\right),$$

where the last inequality comes from the fact that *Q* is an element of Ω. □

We now return to the optimization problem in Equation (27). Given Theorem A1 and the definition of the Blackwell order in Equation (23), it can be rewritten as

(A7) $$\begin{array}{cc} I_{\cap }^{≺}\left(X_{1};\ldots ;X_{n}\;\to \;Y\right)= & \max_{\kappa_{Q|Y},\kappa_{Q|X_{1},\ldots ,}\kappa_{Q|X_{n}}}I_{\kappa }\left(Q,Y\right)\\ & \mathrm{such}\;\mathrm{that}\;\forall i,y,x_{i}\;:\sum_{x_{i}}\kappa_{Q|X_{i}}\left(q|x_{i}\right)P_{X_{i}|Y}\left(x_{i}|y\right)=\kappa_{Q|Y}\left(q|y\right). \end{array}$$

where the optimization is over channels with Q of cardinality $\left(\sum_{i}\left|X_{i}\right|\right)-n+1$. The notation $I_{\kappa }\left(Q;Y\right)$ indicates the mutual information that arises from the marginal distribution $P_{Y}$ and the conditional distribution $\kappa_{Q|Y}$,

$$I_{\kappa }\left(Q;Y\right)=\sum_{y}P_{Y}\left(y\right)\kappa_{Q|Y}\left(q|y\right)\ln\frac{\kappa_{Q|Y}\left(q|y\right)}{\sum_{y^{\prime }}\kappa_{Q|Y}\left(q|y^{\prime }\right)P_{Y}\left(y^{\prime }\right)}$$

Equation (A7) involves maximizing a convex function over the convex polytope defined by the following system of linear (in)equalities:

$$\begin{array}{c} \Lambda =\{(\kappa_{Q|Y},\kappa_{Q|X_{1},\ldots ,}\kappa_{Q|X_{n}}): \end{array}$$

(A8) $$\begin{array}{cc} \forall i,x_{i},q\; & \kappa_{Q|X_{i}}\left(q|x_{i}\right)\geq 0, \end{array}$$

(A9) $$\begin{array}{cc} \forall q,y\; & \kappa_{Q|Y}\left(q|y\right)\geq 0, \end{array}$$

(A10) $$\begin{array}{cc} \forall y\; & \sum_{q}\kappa_{Q|Y}\left(q|y\right)=1, \end{array}$$

(A11) $$\begin{array}{cc} \forall i,x_{i}\; & \sum_{q}\kappa_{Q|X_{i}}\left(q|x_{i}\right)=1, \end{array}$$

(A12) $$\begin{array}{cc} \forall i,y,q\in Q\setminus \{0\}\; & \left[\sum_{x_{i}}\kappa_{Q|X_{i}}\left(q|x_{i}\right)P_{X_{i}Y}\left(x_{i},y\right)\right]-\kappa_{Q|Y}\left(q|y\right)P_{Y}\left(y\right)=0\}, \end{array}$$

We do not place a constraint on q=0 in Equation (A12) because that would be redundant with the constraints Equations (A10) and (A11). Also, note that we replaced the sup in Equation (27) with max in Equation (A7), which is justified since we are optimizing over a finite dimensional, closed, and bounded region (thus the supremum is always achieved).

The maximum of a convex function over a convex polytope is found at one of the vertices of the polytope. To find the solution to Equation (A7), we use a computational geometry package to enumerate the vertices of Λ. We evaluate $I_{\kappa }\left(Y;Q\right)$ at each vertex, and pick the maximum value. This procedure also finds optimal conditional distributions $\kappa_{Q|Y},\kappa_{Q|X_{1},\ldots ,}\kappa_{Q|X_{n}}$. Code is available at [64].

## Appendix D. Continuity of $I_{\cap }^{≺}$

To prove the continuity of $I_{\cap }^{≺}$, we begin by considering the feasible set of the optimization problem in Equation (A7), as specified by the system (in)equalities in Equations (A8) to (A12). For convenience, write this system of (in)equalities in matrix notation,

(A13) $$\Lambda =\left\{\vec{\kappa }\in R^{\left|Q||Y\right|+\sum_{i}\left|Q|\right|X_{i}|}:\kappa \geq 0,A\kappa =a\right\},$$

where $\vec{\kappa }$ is a vector representation of $\left(\kappa_{Q|Y},\kappa_{Q|X_{1},\ldots ,}\kappa_{Q|X_{n}}\right)$, the matrix *A* encodes the left-hand side of Equations (A10) to (A12), and the vector *a* is filled with 1s and 0s, as appropriate.

We first prove the following lemma.

**Lemma A1.** 
*The matrix A defined in Equation *(A13)* is full rank if n−1 or more of the pairwise conditional distributions have $\mathrm{rank}\;P_{Y|X_{i}}=\left|Y\right|$.*

**Proof.** Without loss of generality, assume that $P_{Y}$ has full support (otherwise none of the pairwise marginals $P_{X_{i}Y}$ can achieve rank |Y|). Write *A* in block matrix form as $A=\left[\begin{array}{c} B\\ C \end{array}\right]$, where the matrix *B* has $\left|Y\right|+\sum_{i}\left|X_{i}\right|$ rows and encodes the constraints of Equations (A10) and (A11), and the matrix *C* has n|Y|(|Q|−1) rows and encodes the constraints of Equation (A12). Each row in *B* has a 1 in some column which is zero in every other row of *B* and every row of *C*. This column corresponds either to $\kappa_{Q|Y}\left(0|y\right)$ for a particular *y* (for constraints like Equation (A10)), or to $\kappa_{Q|X_{i}}\left(0|x_{i}\right)$ for a particular *i* and $x_{i}$ (for constraints like Equation (A11)). These columns are 0 in *C* because q=0 is omitted Equation (A12). This means that no row of *B* is a linear combination of other rows in *B* or *C*, and that no row in *C* is a linear combination of any set of other rows that includes a row in *B*. Therefore, if the rows of *A* are linearly dependent, it must be that the rows of *C* are linearly dependent. Next, let ${\vec{c}}^{i,y,q}$ indicate the row of *C* that represents the constraints in Equation (A12) for some source *i* and outcomes y,q≠0. Any such row has a column for each $x_{i}\in X_{i}$ with value $P_{X_{i}Y}\left(x_{i},y\right)$ (at the same index as the row in $\vec{\kappa }$ that represents $\kappa_{Q|X_{i}}\left(q|x_{i}\right)$). Since $P_{X_{i}Y}\left(x_{i},y\right)>0$ for at least one $x_{i}\in X_{i}$, one of these columns must be non-zero. At the same time, these columns are zero in every row ${\vec{c}}^{j,y,q^{\prime }}$ where j≠i or $q^{\prime }\neq q$. This means that row ${\vec{c}}^{i,y,q}$ can only be a linear combination of other rows in *C* if, for all $x_{i}$, $P_{X_{i}Y}\left(x_{i},y\right)$ is a linear combination of $P_{X_{i}Y}\left(x_{i},y^{\prime }\right)$ for $y^{\prime }\neq y$. In linear algebra terms, this can be stated as $\mathrm{rank}\;P_{Y|X_{i}}<\left|Y\right|$. The previous argument shows that if *A* is linearly dependent, there must be at least one source *i* with $\mathrm{rank}\;P_{Y|X_{i}}<\left|Y\right|$ and some row ${\vec{c}}^{i,y,q}$ which is a linear combination of other rows from *C*. Observe that this row ${\vec{c}}^{i,y,q}$ has a column with value $P_{Y}\left(y\right)>0$ (at the same index as the row in $\vec{\kappa }$ that represents $\kappa_{Q|Y}\left(q|y\right)$). This column is zero in every other row ${\vec{c}}^{i,y^{\prime },q^{\prime }}$ for $y^{\prime }\neq y$ or $q^{\prime }\neq q$. This means that ${\vec{c}}^{i,y,q}$ is a linear combination of a set of other rows in *C* that include some row ${\vec{c}}^{j,y,q}$ for j≠i. This implies that ${\vec{c}}^{j,y,q}$ is also a linear combination of other rows in *C*, which means that $\mathrm{rank}\;P_{Y|X_{j}}<\left|Y\right|$. We have shown that if *A* is linearly dependent, there must be at least two pairwise conditionals with $\mathrm{rank}\;P_{Y|X_{i}}<\left|Y\right|$. □

We are now ready to prove Theorem 5.

**Proof of Theorem 5.** For the case of a single source (n=1), $I_{\cap }^{≺}$ reduces to the mutual information $I_{\cap }^{≺}=I\left(Y;X_{1}\right)$, which is continuous (Section 2.3, [75]). Thus, without loss of generality, we assume that n≥2. Next, we define some notation. Note that the optimum value ($I_{\cap }^{≺}$) and the feasible set of the optimization problem in Equation (A7) is a function of the pairwise marginal distributions $P_{X_{1}Y},\ldots ,P_{X_{n}Y}$. We write Ω for the set of all pairwise marginal distributions which have the same marginal over *Y*:

$$\Omega =\left\{\left(q_{X_{1}Y},\ldots ,q_{X_{n}Y}\right):\sum_{x_{i}}q_{X_{i}Y}\left(x_{i},y\right)=\sum_{x_{j}}q_{X_{j}Y}\left(x_{j},y\right)\;\;\forall i,j\right\}.$$

For any r∈Ω, let $I_{\cap }^{≺}\left(r\right)$ indicate the corresponding optimum value in Equation (A7), given the marginals in *r*, and let Λ(r) indicate the feasible set of the optimization problem, as defined in Equation (A13). Note that the matrix *A* in Equation (A13) depends on the choice of *r*, which we indicate by writing it as the matrix-valued function A(r). Given any $r=(q_{X_{1}Y},\ldots ,q_{X_{n}Y})\in \Omega$ and feasible solution $\kappa =\left(\kappa_{Q|Y},\kappa_{Q|X_{1},\ldots ,}\kappa_{Q|X_{n}}\right)\in \Lambda \left(r\right)$, let I(r,κ) indicate the corresponding mutual information I(Q;Y), where the marginal distribution over *Y* is specified by *r* and the conditional distribution of *Q* given *Y* is specified by $\kappa_{Q|Y}$. Using this notation, $I_{\cap }^{≺}\left(r\right)=\max_{\kappa \in \Lambda (r)}I\left(r,\kappa \right)$. Below, we show that $I_{\cap }^{≺}\left(r\right)$ is continuous if *r* is *rank regular* [76], which means that there is a neighborhood U⊆Ω of *r* such that $\mathrm{rank}\;A\left(r^{\prime }\right)=\mathrm{rank}\;A\left(r\right)$ for all $r^{\prime }\in U$. Then, to prove the theorem, we assume that A(r) is full rank. Given Lemma A1, this is true as long as n−1 or more of the pairwise conditionals $P_{Y|X_{i}}$ have $\mathrm{rank}\;P_{Y|X_{i}}=\left|Y\right|$. Note that a matrix *M* is full rank iff the singular values σ(M) are all strictly positive. Since A(r) is full rank, and A(r) and σ(M) are continuous, there is a neighborhood *U* of *r* such that the singular values $\sigma (A\left(r^{\prime }\right))$ are all strictly positive for all $r^{\prime }\in U$, therefore all $A(r^{\prime }))$ have full rank. This shows that *r* is rank regular and so $I_{\cap }^{≺}$ is continuous at *r*. We now prove that $I_{\cap }^{≺}\left(r\right)$ is continuous if A(r) is rank regular. To do so, we will use Hoffman’s Theorem [77,78]. In our case, it states that for any pair of marginals $r,r^{\prime }\in \Omega$ and a feasible solution $\kappa^{\prime }\in \Lambda \left(r^{\prime }\right)$, there exists a feasible solution κ∈Λ(r) such that

(A14) $$∥\kappa -\kappa^{\prime }∥\leq \alpha ∥A\left(r\right)-A\left(r^{\prime }\right)∥,$$

where α is a constant that does not depend on $r^{\prime }$ or $\kappa^{\prime }$. (In the notation of [78], we take $G=G^{\prime }$, $g=g^{\prime }$ and $d^{\prime }=d$, and use that the norm of $s=\kappa^{\prime }$ is bounded, given that it is finite dimensional and has entries in [0,1]). We will also use Daniel’s theorem (Theorem 4.2, [78]), which states that for any $r,r^{\prime }\in \Omega$ such that $\mathrm{rank}\;A\left(r\right)=\mathrm{rank}\;A\left(r^{\prime }\right)$, and any feasible solution κ∈Λ(r), there exists $\kappa^{\prime }\in \Lambda \left(r^{\prime }\right)$ such that

(A15) $$∥\kappa -\kappa^{\prime }∥\leq \beta ∥A\left(r\right)-A\left(r^{\prime }\right)∥,$$

where β is a constant that doesn’t depend on $r^{\prime }$ (in the notation of [78], $\varepsilon^{\prime }=∥A\left(r\right)-A\left(r_{i}^{\prime }\right)∥$ and again use that κ have a bounded norm). Now consider also any sequence $r_{1}^{\prime },r_{2}^{\prime },\ldots \in \Omega$ that converges to a marginal r∈Ω. Let $\kappa_{i}^{\prime }\in \Lambda \left(r_{i}^{\prime }\right)$ indicate an optimal solution of Equation (A7) for $r_{i}$, so that $I\left(r_{i}^{\prime },\kappa_{i}^{\prime }\right)=I_{\cap }^{≺}\left(r_{i}^{\prime }\right)$. Given Equation (A14), there is a corresponding sequence $\kappa_{1},\kappa_{2},\ldots \in \Lambda \left(r\right)$ such that

$$∥\kappa_{i}-\kappa_{i}^{\prime }∥\leq \alpha ∥A\left(r\right)-A\left(r_{i}^{\prime }\right)∥.$$

Since A(·) is continuous and $r_{i}^{\prime }$ converges to *r*, we have $\lim_{i\to \infty }A\left(r_{i}^{\prime }\right)=A\left(r\right)$ and therefore $\lim_{i\to \infty }∥\kappa_{i}-\kappa_{i}^{\prime }∥=0$. This implies

(A16) $$0=\lim_{i\to \infty }I\left(r_{i}^{\prime },\kappa_{i}^{\prime }\right)-I\left(r,\kappa_{i}\right)\geq \lim_{i\to \infty }I_{\cap }^{≺}\left(r_{i}^{\prime }\right)-I_{\cap }^{≺}\left(r\right)$$

where we first used continuity of mutual information, $I\left(r_{i}^{\prime },\kappa_{i}^{\prime }\right)=I_{\cap }^{≺}\left(r_{i}^{\prime }\right)$ and $I\left(r,\kappa_{i}\right)\leq I_{\cap }^{≺}\left(r\right)$. Now assume that *r* is rank regular. Since $r_{i}$ converges to *r*, $\mathrm{rank}\;A\left(r_{i}^{\prime }\right)=\mathrm{rank}\;A\left(r\right)$ for all sufficiently large *i*. Let κ∈Λ(r) be an optimal solution of Equation (A7) for *r*, so that $I\left(r,\kappa \right)=I_{\cap }^{≺}\left(r\right)$. Given Equation (A15), for all sufficiently large *i* there exists $\kappa_{i}^{\prime }\in \Lambda \left(r_{i}^{\prime }\right)$ such that

$$∥\kappa -\kappa_{i}^{\prime }∥\leq \beta ∥A\left(r\right)-A\left(r_{i}^{\prime }\right)∥.$$

As before, we have $\lim_{i\to \infty }A\left(r_{i}^{\prime }\right)=A\left(r\right)$ and $\lim_{i\to \infty }∥\kappa -\kappa_{i}^{\prime }∥=0$, which implies

(A17) $$0=\lim_{i\to \infty }I\left(r_{i}^{\prime },\kappa_{i}^{\prime }\right)-I\left(r,\kappa \right)\leq \lim_{i\to \infty }I_{\cap }^{≺}\left(r_{i}^{\prime }\right)-I_{\cap }^{≺}\left(r\right)$$

where we first used continuity of mutual information, $I\left(r_{i}^{\prime },\kappa_{i}^{\prime }\right)\leq I_{\cap }^{≺}\left(r_{i}^{\prime }\right)$, and $I\left(r,\kappa \right)\leq I_{\cap }^{≺}\left(r\right)$. Combining Equations (A16) and (A17) proves continuity, $\lim_{i\to \infty }I_{\cap }^{≺}\left(r_{i}\right)=I_{\cap }^{≺}\left(r\right)$, under the assumption that A(r) is rank regular. Finally, note that A(r) is a real analytic function of *r*. This means that almost all *r* rank regular, because those *r* which are not rank regular form a proper analytic subset of Ω (which has measure zero) [76]. Thus, $I_{\cap }^{≺}\left(r\right)$ is continuous almost everywhere. □

We finish our analysis of the continuity of $I_{\cap }^{≺}$ by showing global continuity when the target is a binary random variable.

**Corollary A1.** 
*$I_{\cap }^{≺}\left(X_{1};\ldots ;X_{n}\;\to \;Y\right)$ is continuous everywhere when Y is a binary random variable.*

**Proof.** In an overloading of notation, let $I_{\cap }^{≺}\left(r\right)$ and $I_{r}\left(X_{i};Y\right)$ indicate $I_{\cap }^{≺}\left(X_{1};\ldots ;X_{n}\;\to \;Y\right)$ and the mutual information $I(X_{i};Y)$, respectively, for the joint distribution $r_{X_{1}\ldots X_{n}Y}$. By Theorem 5, $I_{\cap }^{≺}$ can only be discontinuous at the joint distribution $P_{X_{1}\ldots X_{n}Y}$ if there is a source $X_{i}$ with $\mathrm{rank}\;P_{Y|X_{i}}=1<\left|Y\right|$. However, if source $X_{i}$ has rank 1, then the conditional distributions $P_{Y|X_{i}=x_{i}}$ are the same for all $x_{i}$, so $I_{P}\left(X_{i};Y\right)=0$ and $I_{\cap }^{≺}\left(P\right)=0$ (since $0\leq I_{\cap }^{≺}\left(P\right)\leq I_{P}\left(X_{i};Y\right)$). Finally, consider any sequence of joint distributions $s_{X_{1}\ldots X_{n}Y}^{\left(n\right)}$ for n=1,2,… that converges to $P_{X_{1}\ldots X_{n}Y}$. We have

$$0\leq \lim_{n\to \infty }I_{\cap }^{≺}\left(s^{\left(n\right)}\right)\leq \lim_{n\to \infty }I_{s^{\left(n\right)}}\left(X_{i};Y\right)=I_{P}\left(X_{i};Y\right)=0,$$

where we used the continuity of mutual information. This shows that $\lim_{n\to \infty }I_{\cap }^{≺}\left(s^{\left(n\right)}\right)=0=I_{\cap }^{≺}\left(P\right)$, proving continuity. □

## Appendix E. Behavior of $I_{\cap }^{≺}$ on Gaussian Random Variables

Although in this paper we focused on random variables with finite sets of outcomes, we can briefly comment on the behavior of Blackwell redundancy on Gaussian random variables. Suppose that all sources $X_{1},\ldots ,X_{n}$ and the target *Y* are continuous-valued, and that the pairwise marginals $P_{X_{i}Y}$ are multivariate Gaussians. In addition, suppose that *Y* is one-dimensional (the sources $X_{i}$ can be multi-dimensional). Given these assumptions, Barrett [51] analyzed the $I_{\cup }^{\mathrm{BROJA}}$ measure and showed that the corresponding excluded information obeys $E(X_{j}\;\to \;Y|X_{i};X_{j})=0$ whenever $I\left(X_{i};Y\right)\leq I\left(X_{j};Y\right)$. Recall that $I_{\cup }^{\mathrm{BROJA}}$ is equivalent to Blackwell union information $I_{\cup }^{≺}$. Then, given the Blackwell property, Theorem 4, and the data processing inequality, Equation (24), the result in Ref. [51] implies that $X_{i}{≺}_{Y}X_{j}$ if and only if $I\left(X_{i};Y\right)\leq I\left(X_{j};Y\right)$. Thus, for Gaussian random variables, Blackwell redundancy $I_{\cap }^{≺}$ is equivalent to $I_{\cap }^{\mathrm{MMI}}$ redundancy, as defined in Equation (19). This parallels the case for most other redundancy measures [51].

## Appendix F. Operational Interpretation of the $I_{\cap }^{\mathrm{GH}}$

As mentioned in the main text, the redundancy measure $I_{\cap }^{\mathrm{GH}}$ is a special case of Equation (7), where the “more informative” order B⊏C is defined in terms of conditional independence B−C−Y. Here we show that this ordering relation can be given an operational interpretation, which is similar but distinct from the operational interpretation of the Blackwell order ${≺}_{Y}$ discussed in Section 5.1.

To introduce this operational interpretation, let the random variable *Y* represent the state of the environment, and assume there are two random variables *B* and *C* which have some information about *Y*. Suppose that an agent tries to maximize expected utility u(a,y) by using a strategy that depends either on the outcomes of *B* or *C*. Blackwell’s theorem tells us that $B{≺}_{Y}C$ iff an agent with access to *C* can always achieve higher expected utility than an agent with access to *B*. It is possible, however, the agent with access to *C* may do worse than the agent with access to *B*, *conditional on the event that random variable C has some particular outcome c*. In the following theorem, we show B−C−Y iff the agent cannot do better with *B* than *C*, even when conditioned on any particular outcome C=c. (We thank Johannes Rauh for suggesting this simplified proof).

**Theorem A2.** 
*Given random variables B, C, and Y, B−C−Y if and only if*

(A18)
$$\max_{\kappa_{A|B}}\sum_{y,a,b}P_{YB|C}\left(y,b|c\right)\kappa_{A|B}\left(a|b\right)u\left(a,y\right)\leq \max_{\kappa_{A|C}}\sum_{y,a}P_{Y|C}\left(y|c\right)\kappa_{A|C}\left(a|c\right)u\left(a,y\right).$$

*for all utility functions u(a,y) and all c∈C with $P_{C}\left(c\right)>0$.*

**Proof.** Consider any c∈C with $P_{C}\left(c\right)>0$. By multiplying both sides of Equation (A18) by $P_{C}\left(c\right)$ and rearranging, this inequality can be rewritten as

(A19) $$\begin{array}{c} \max_{\kappa_{A|B}}\sum_{y,a,b}P_{Y}\left(y\right)P_{C|Y}\left(c|y\right)P_{B|Y,C}\left(b|y,c\right)\kappa_{A|B}\left(a|b\right)u\left(a,y\right)\\ \;\;\;\;\;\leq \max_{\kappa_{A|C}}\sum_{y,a}P_{Y}\left(y\right)P_{C|Y}\left(c|y\right)\kappa_{A|C}\left(a|c\right)u\left(a,y\right). \end{array}$$

Note that if Equation (A18) holds for a given *c* and all utility functions, then it must also hold for the utility function $u^{\prime }\left(a,y\right):=P_{C|Y}\left(c|y\right)u\left(a,y\right)$. Plugging into Equation (A19) gives

(A20) $$\begin{array}{c} \max_{\kappa_{A|B}}\sum_{y,a,b}P_{Y}\left(y\right)P_{B|Y,C}\left(b|y,c\right)\kappa_{A|B}\left(a|b\right)u^{\prime }\left(a,y\right)\leq \max_{\kappa_{A|C}}\sum_{y,a}P_{Y}\left(y\right)\kappa_{A|C}\left(a|c\right)u^{\prime }\left(a,y\right). \end{array}$$

Now define two random variables: a constant random variable ${\hat{C}}_{c}$ with a single outcome *c* and ${\hat{B}}_{c}$ with the same outcomes as *B* but having the conditional distribution $P_{{\hat{B}}_{c}|Y}=P_{B|Y,C=c}$. Then, Equation (A20) can be written in terms of these random variables as

(A21) $$\max_{\kappa_{A|B}}\sum_{y,a,b}P_{Y}\left(y\right)P_{{\hat{B}}_{c}|Y}\left(b|y\right)\kappa_{A|B}\left(a|b\right)u^{\prime }\left(a,y\right)\leq \max_{\kappa_{A|C}}\sum_{y,a}P_{Y}\left(y\right)P_{{\hat{C}}_{c}|Y}\left(c|y\right)\kappa_{A|C}\left(a|c\right)u^{\prime }\left(a,y\right).$$

Given Equations (25) and (26), Equation (A21) holds for all $u^{\prime }$ iff ${\hat{B}}_{c}{≺}_{Y}{\hat{C}}_{c}$. Since ${\hat{C}}_{c}$ has a single outcome, it is independent of *Y*. That means ${\hat{B}}_{c}$ must be also independent of *Y* and so $P_{{\hat{B}}_{c}|Y=y}=P_{B|Y=y,C=c}$ is the same for all *y*, implying that $P_{B|Y=y,C=c}=P_{B|C=c}$. Since this holds for all c∈C, $P_{B|YC}=P_{B|C}$ and therefore B−C−Y. □

Given Theorem A2, $I_{\cap }^{\mathrm{GH}}$ can be given the following operational interpretation. Imagine two agents, Alice and Bob, who can acquire information about *Y* via different random variables, and then use this information to maximize their expected utility. Suppose that Alice has access to one of the sources $X_{i}$. Then, $I_{\cap }^{\mathrm{GH}}$ is the maximum information that Bob can have about *Y* without being able to do better than Alice on any utility function, regardless of which source $X_{i}$ Alice has access to, and even when conditioned on $X_{i}$ having any particular outcome $x_{i}$.

## Appendix G. Equivalence of $I_{\cup }^{≺}$ and $I_{\cup }^{\mathrm{BROJA}}$

The following proves that $I_{\cup }^{≺}$ and $I_{\cup }^{\mathrm{BROJA}}$, as defined via the optimization problems in Equations (28) and (31), are equivalent.

**Theorem A3.** 

$I_{\cup }^{≺}\left(X_{1};\ldots ;X_{n}\;\to \;Y\right)=I_{\cup }^{\mathrm{BROJA}}\left(X_{1};\ldots ;X_{n}\;\to \;Y\right).$

**Proof.** Let ${\tilde{X}}_{1},\ldots ,{\tilde{X}}_{n}$ be a set of random variables that achieve $I\left(Y;{\tilde{X}}_{1},\ldots ,{\tilde{X}}_{n}\right)=I_{\cup }^{\mathrm{BROJA}}$$\left(X_{1};\ldots ;X_{n}\;\to \;Y\right)$. Define the random variable $Q:=({\tilde{X}}_{1},\ldots ,{\tilde{X}}_{n})$, and note that ${\tilde{X}}_{i}{≺}_{Y}Q$ for all *i*. Since $P_{{\tilde{X}}_{i}Y}=P_{X_{i}Y}$ for all *i*, it must be that $X_{i}{≺}_{Y}Q$ for all *i*. Thus *Q* satisfies the constraints of the optimization problem in Equation (28), so

(A22) $$I_{\cup }^{≺}\left(X_{1};\ldots ;X_{n}\;\to \;Y\right)\leq I\left(Y;Q\right)=I_{\cup }^{\mathrm{BROJA}}\left(X_{1};\ldots ;X_{n}\;\to \;Y\right).$$

Next, consider the optimization in Equation (28). For any ϵ>0, let *Q* be a random variable that satisfies $X_{i}{≺}_{Y}Q$ and achieves

(A23) $$I\left(Y;Q\right)\leq I_{\cup }^{≺}\left(X_{1};\ldots ;X_{n}\;\to \;Y\right)+\epsilon .$$

For each *i*, let $\kappa_{X_{i}|Q}$ be a channel that obeys $P_{X_{i}|Y}\left(x_{i}|y\right)=\sum_{q}\kappa_{X_{i}|Q}\left(x_{i}|q\right)P_{Q|Y}\left(q|y\right)$ (such a channel must exist since $X_{i}{≺}_{Y}Q$). Define the random variables ${\tilde{X}}_{1},\ldots ,{\tilde{X}}_{n}$ with the joint distribution

(A24) $$P_{YQ{\tilde{X}}_{1}\ldots {\tilde{X}}_{n}}\left(y,q,x_{1},\ldots ,x_{n}\right)=P_{Y}\left(y\right)P_{Q|Y}\left(q|y\right)\prod_{i}\kappa_{X_{i}|Q}\left(x_{i}|q\right).$$

Note that the pairwise marginals obey $P_{{\tilde{X}}_{i}Y}=P_{X_{i}Y}$. Thus, all of the ${\tilde{X}}_{i}$ satisfy the marginal constraints in the right hand side of Equation (31), so

(A25) $$I_{\cup }^{\mathrm{BROJA}}\left(X_{1};\ldots ;X_{n}\;\to \;Y\right)\leq I\left(Y;{\tilde{X}}_{1},\ldots ,{\tilde{X}}_{n}\right).$$

By elementary properties of mutual information, we have

(A26) $$I\left(Y;{\tilde{X}}_{1},\ldots ,{\tilde{X}}_{n}\right)\leq I\left(Y;Q,{\tilde{X}}_{1},\ldots ,{\tilde{X}}_{n}\right)$$

Given Equation (A24), the Markov condition $Y-Q-{\tilde{X}}_{1},\ldots ,{\tilde{X}}_{n}$ holds, so

(A27) $$I\left(Y;{\tilde{X}}_{1},\ldots ,{\tilde{X}}_{n}\right)\leq I\left(Y;Q\right)$$

by the data processing inequality. Combining Equations (A23) and (A25) to (A27) implies

$$I_{\cup }^{\mathrm{BROJA}}\left(X_{1};\ldots ;X_{n}\;\to \;Y\right)\leq I\left(Y;Q\right)\leq I_{\cup }^{≺}\left(X_{1};\ldots ;X_{n}\;\to \;Y\right)+\epsilon .$$

Since this holds for all ϵ, we can take the limit ϵ→0 to give $I_{\cup }^{\mathrm{BROJA}}\left(X_{1};\ldots ;X_{n}\;\to \;Y\right)\leq I_{\cup }^{≺}\left(X_{1};\ldots ;X_{n}\;\to \;Y\right)$. The result follows by combining with Equation (A22). □

## Appendix H. Relation between $I_{\cap }^{\mathrm{WB}}$ and Our General Framework

Here we consider whether the redundancy measure $I_{\cap }^{\mathrm{WB}}$ proposed by Williams and Beer [11] can be put in the general form Equation (7). This measure is defined as

(A28) $$I_{\cap }^{\mathrm{WB}}\left(X_{1};\ldots ;X_{n}\;\to \;Y\right):=\sum_{y}P_{Y}\left(y\right)\min_{i}I\left(X_{i};Y=y\right),$$

where $I(X_{i};Y=y)$ is called the “specific information” between $X_{i}$ and target outcome *y*,

$$I\left(X_{i};Y=y\right):=D_{\mathrm{KL}}\left(P_{X_{i}|Y=y}∥P_{X_{i}}\right)=\sum_{x_{i}}P_{X_{i}|Y}\left(x_{i}|y\right)\log\frac{P_{X_{i}|Y}\left(x_{i}|y\right)}{P_{X_{i}}\left(x_{i}\right)},$$

and $D_{\mathrm{KL}}$ is Kullback-Leibler (KL) divergence.

Specific information obeys $I\left(X;Y\right)=\sum_{y}P_{Y}\left(y\right)I\left(X;Y=y\right)$. Thus, Equation (A28) looks similar to a mutual information expression, where each specific information term is given by the smallest specific information that *y* carries about any of the sources. Motivated by this interpretation, one might ask whether there exists a random variable *Q* whose specific information terms are equal to $I\left(Q;Y=y\right)=\min_{i}I\left(X_{i};Y=y\right)$ for each *y*. If such a random variable existed, then $I_{\cap }^{\mathrm{WB}}$ could be written as

(A29) $$I_{\cap }^{\mathrm{WB}}\left(X_{1};\ldots ;X_{n}\;\to \;Y\right)\overset{?}{=}\max_{Q}I\left(Q;Y\right)\;\mathrm{such}\;\mathrm{that}\;\forall i,y:I\left(Q;Y=y\right)\leq I\left(X_{i};Y=y\right),$$

which has the form of Equation (7), with the ⊏ order defined as

(A30) A⊏B iff I(A;Y=y)≤I(B;Y=y) for all y∈Y.

Here we provide a counterexample to demonstrate that such a variable does not exist in general, and so therefore Equation (A29) is not generally valid. Suppose *Y* has three outcomes Y={0,1,2} with a uniform distribution, and consider two binary sources $X_{1},X_{2}$ with the following conditional distributions,

$$\begin{array}{cc} P_{X_{1}|Y}\left(x_{1}|y\right) & =\left\{\begin{array}{cc} \delta (x_{1},y) & \mathrm{if}\;y\in \{0,1\}\\ \frac{1}{2}\delta \left(x_{1},0\right)+\frac{1}{2}\delta \left(x_{1},1\right) & \mathrm{if}\;y=2 \end{array}\right.\\ P_{X_{2}|Y}\left(x_{1}|y\right) & =\left\{\begin{array}{cc} \delta (x_{1},y) & \mathrm{if}\;y\in \{0,2\}\\ \frac{1}{2}\delta \left(x_{1},0\right)+\frac{1}{2}\delta \left(x_{1},2\right) & \mathrm{if}\;y=1 \end{array}\right. \end{array}$$

In this case, a simple calculation shows that the specific information obeys (in bits)

$$\begin{array}{cc} I(X_{1};Y=0)=1 & I(X_{2};Y=0)=1\\ I(X_{1};Y=1)=1 & I(X_{2};Y=1)=0\\ I(X_{1};Y=2)=0 & I(X_{2};Y=2)=1 \end{array}$$

Plugging into Equation (A28) gives $I_{\cap }^{\mathrm{WB}}\left(X_{1};X_{2}\;\to \;Y\right)=1/3$.

Now consider the optimization problem in Equation (A29). Since $I\left(X_{1};Y=2\right)=I\left(X_{2};Y=1\right)=0$, any allowed *Q* must satisfy I(Q;Y=1)=I(Q;Y=2)=0 and therefore $P_{Q|Y=1}=P_{Q}=P_{Q|Y=2}$. Combined with the marginalization identity $P_{Y}\left(0\right)P_{Q|Y=0}+P_{Y}\left(1\right)P_{Q|Y=1}+P_{Y}\left(2\right)P_{Q|Y=2}=P_{Q}$, this implies that $P_{Q|Y=0}=P_{Q}$ and therefore that I(Q;Y=0)=0. Thus, any allowed *Q* obeys $I\left(Q;Y\right)=0\neq I_{\cap }^{\mathrm{WB}}$. This means that $I_{\cap }^{\mathrm{WB}}$ cannot be expressed in the form of Equation (7) when ⊏ is defined as Equation (A30).

## Appendix I. Miscellaneous Derivations

**Proof of Lemma 1.** We use a modified version of the example in [39,68]. Consider a set of n≥3 sources. The inclusion-exclusion principle states that

(A31) $$I_{\cup }\left(X_{1};\ldots ;X_{n}\;\to \;Y\right)=\sum_{J\subseteq \{1,\ldots ,n\}\setminus \{⌀\}}{\left(-1\right)}^{\left|J\right|-1}I_{\cap }\left(X_{J_{1}};\ldots ;X_{J_{\left|J\right|}}\;\to \;Y\right).$$

Now, let $X_{1},\ldots ,X_{n-1}$ be uniformly distributed and statistically independent binary random variables, and take $X_{n}=X_{1}\;\mathrm{XOR}\;X_{2}$ and $Y=(X_{1},X_{2},X_{n})$. Note that $I(Y;X_{i})=1\;\mathrm{bit}$ for i∈{1,2,n} and $I(Y;X_{i})=0$ for i∈{3,…,n−1}, and that $I(Y;X_{1},\ldots ,X_{n})=2\;\mathrm{bit}$. Thus, $I_{\cap }\left(X_{i};X_{j}\;\to \;Y\right)=0$ whenever i∈{3,…,n−1} or j∈{3,…,n−1}, as follows from *Symmetry*, *Self-redundancy*, and *Monotonicity*. Note also that the outcomes of *Y* are simply a relabelling of $\left(X_{1},X_{2}\right)$, and similarly for $\left(X_{1},X_{n}\right)$ and $\left(X_{2},X_{n}\right)$. Then, since by *Independent identity* property, $I_{\cap }\left(X_{i};X_{j}\;\to \;Y\right)=0$ for i≠j where i,j∈{1,2,n}. Thus, $I_{\cap }\left(X_{i};X_{j}\;\to \;Y\right)=0$ for all pairs i≠j. By *Monotonicity*, redundancy is 0 for any set of 2 or more sources. Plugging this into Equation (A31) gives

$$I_{\cup }\left(X_{1};\ldots ;X_{n}\;\to \;Y\right)=\sum_{i}I_{\cap }\left(X_{i}\;\to \;Y\right)=\sum_{i}I\left(X_{i}\;\to \;Y\right)=3\;\mathrm{bit}$$

Note that this exceeds the total amount of information about the target provided jointly by all sources, which is only 2 bits, so $I_{\cup }\left(X_{1};\ldots ;X_{n}\;\to \;Y\right)\neg \leq I\left(Y;X_{1},\ldots ,X_{n}\right)$. □

**Proof of Theorem 3.** Without loss of generality, let i=1. We will use that $U^{≺}\left(X_{1}\;\to \;Y|X_{1};\ldots ;X_{n}\right)=0$ is equivalent to

(A32) $$I_{\cap }^{≺}\left(X_{1};\ldots ;X_{n}\;\to \;Y\right)=I\left(X_{1};Y\right).$$

We will use that by monotonicity of mutual information with respect to ${≺}_{Y}$ (see Section 4.1),

(A33) $$I\left(X_{1};Y\right)\geq I_{\cap }^{≺}\left(X_{1};\ldots ;X_{n}\;\to \;Y\right).$$

We first prove the “if” direction. Since $Q=X_{1}$ is in the feasible set of Equation (27), $I_{\cap }^{≺}\left(X_{1};\ldots ;X_{n}\;\to \;Y\right)\geq I\left(X_{1};Y\right)$. Combining with Equation (A33) gives Equation (A32). We now prove the “only if” direction. As described in Appendix C, $I_{\cap }^{≺}$ can be expressed as an optimization over a finite dimensional, closed, and bounded region, so the supremum in Equation (27) is achieved. Thus, there is some *Q* such that $Q{≺}_{Y}X_{i}$ for all *i* and

(A34) $$\begin{array}{cc} I(Y;Q) & =I_{\cap }^{≺}\left(X_{1};\ldots ;X_{n}\;\to \;Y\right). \end{array}$$

Since $Q{≺}_{Y}X_{1}$, there is a conditional probability distribution $\kappa_{Q|X_{1}}$ such that $P_{Q|Y}\left(q|y\right)=\sum_{x_{1}}\kappa_{Q|X_{1}}\left(q|x_{1}\right)P_{X_{1}|Y}\left(x_{1}|y\right)$. Define a random variable $\tilde{Q}$ with the joint distribution

$$P_{YX_{1}\tilde{Q}}\left(y,x_{1},q\right)=\kappa_{Q|X_{1}}\left(q|x_{1}\right)P_{YX_{1}}\left(y,x_{1}\right).$$

We will use that $P_{QY}=P_{\tilde{Q}Y}$. Then, the chain rule for mutual information gives

$$\begin{array}{cc} I(Y;X_{1},\tilde{Q}) & =I\left(Y;\tilde{Q}\right)+I\left(Y;X_{1}|\tilde{Q}\right)=I\left(Y;X_{1}\right)+I\left(Y;\tilde{Q}|X_{1}\right)=I\left(Y;X_{1}\right), \end{array}$$

where we used the Markov condition $Y-X_{1}-\tilde{Q}$. Combining and rearranging gives

(A35) $$I\left(Y;\tilde{Q}\right)=I\left(Y;X_{1}\right)-I\left(Y;X_{1}|\tilde{Q}\right).$$

Now assume that Equation (A32) holds. Combining with Equation (A34) and $P_{QY}=P_{\tilde{Q}Y}$ gives $I\left(Y;X_{1}\right)=I\left(Y;Q\right)=I\left(Y;\tilde{Q}\right)$. Combining with Equation (A35) gives $I(Y;X_{1}|\tilde{Q})=0$, meaning that the Markov condition $Y-\tilde{Q}-X_{1}$ holds and therefore $X_{1}{≺}_{Y}\tilde{Q}$. Since $Q{≺}_{Y}X_{i}$ for all *i* and $P_{QY}=P_{\tilde{Q}Y}$, it also the case that $\tilde{Q}{≺}_{Y}X_{i}$ for all *i*. Finally, since ${≺}_{Y}$ is transitive, $X_{1}{≺}_{Y}X_{i}$ for all *i*, which is the desired result. □

**Proof of Theorem 4.** Without loss of generality, let i=1. We will use that $E^{≺}\left(X_{1}\;\to \;Y|X_{1};\ldots ;X_{n}\right)=0$ is equivalent to

(A36) $$I_{\cup }^{≺}\left(X_{1};\ldots ;X_{n}\;\to \;Y\right)=I\left(X_{1};Y\right).$$

We will use that by monotonicity of mutual information with respect to ${≺}_{Y}$ (see Section 4.1),

(A37) $$I\left(X_{1};Y\right)\leq I_{\cup }^{≺}\left(X_{1};\ldots ;X_{n}\;\to \;Y\right).$$

We first prove the “if” direction. Since $Q=X_{1}$ is in the feasible set of Equation (28), $I_{\cup }^{≺}\left(X_{1};\ldots ;X_{n}\;\to \;Y\right)\leq I\left(X_{1};Y\right)$. Combining with Equation (A37) gives Equation (A36). We now prove the “only if” direction. As we show in Appendix G, $I_{\cup }^{≺}$ is equivalent to $I_{\cup }^{\mathrm{BROJA}}$, which is defined as an optimization over a finite dimensional, closed, and bounded region. Thus the infimum in Equation (28) is always achieved, so there is some *Q* such that $X_{i}{≺}_{Y}Q$ for all *i* and

(A38) $$\begin{array}{cc} I(Y;Q) & =I_{\cup }^{≺}\left(X_{1};\ldots ;X_{n}\;\to \;Y\right). \end{array}$$

Moreover, since $X_{1}{≺}_{Y}Q$, there is a conditional probability distribution $\kappa_{X_{1}|Q}$ such that $P_{X_{1}|Y}\left(x_{1}|y\right)=\sum_{q}\kappa_{X_{1}|Q}\left(x_{1}|q\right)P_{Q|Y}\left(q|y\right)$. Define a random variable ${\tilde{X}}_{1}$ with the joint distribution

$$P_{Y{\tilde{X}}_{1}Q}\left(y,x_{1},q\right)=\kappa_{X_{1}|Q}\left(x_{1}|q\right)P_{QY}\left(q,y\right).$$

We will use that $P_{X_{1}Y}=P_{{\tilde{X}}_{1}Y}$. Then, using the chain rule for mutual information,

$$\begin{array}{cc} I(Y;{\tilde{X}}_{1},Q) & =I\left(Y;{\tilde{X}}_{1}\right)+I\left(Y;Q|{\tilde{X}}_{1}\right)=I\left(Y;Q\right)+I\left(Y;{\tilde{X}}_{1}|Q\right)=I\left(Y;Q\right), \end{array}$$

where we used the Markov condition $Y-Q-{\tilde{X}}_{1}$. Combining and rearranging gives

(A39) $$I\left(Y;{\tilde{X}}_{1}\right)=I\left(Y;Q\right)-I\left(Y;Q|{\tilde{X}}_{1}\right).$$

Now assume that Equation (A36) holds. Combining with Equation (A38) and $P_{X_{1}Y}=P_{{\tilde{X}}_{1}Y}$ gives $I\left(Y;X_{1}\right)=I\left(Y;{\tilde{X}}_{1}\right)=I\left(Y;Q\right)$. Combining with Equation (A39) gives $I(Y;Q|{\tilde{X}}_{1})=0$, meaning that the Markov condition $Y-{\tilde{X}}_{1}-Q$ holds and therefore $Q{≺}_{Y}{\tilde{X}}_{1}$. Since $P_{X_{1}Y}=P_{{\tilde{X}}_{1}Y}$, it is also the case that $Q{≺}_{Y}X_{1}$. Finally, since $X_{i}{≺}_{Y}Q$ for all *i* and ${≺}_{Y}$ is transitive, $X_{i}{≺}_{Y}X_{1}$ for all *i*, which is the desired result. □

**Proof of Theorem 6.** Consider any random variable *Q* which achieves the maximum in Equation (27). This implies there are channels $\kappa_{Q|X_{1}}$ and $\kappa_{Q|X_{2}}$ such that for any q∈Q and $\left(x_{1},x_{2}\right)\in X_{1}\times X_{2}$ with $p_{X_{1}X_{2}}\left(x_{1},x_{2}\right)>0$,

$$\begin{array}{cc} P_{Q|X_{1}X_{2}}\left(q|x_{1},x_{2}\right) & =\sum_{x_{1}^{\prime }}\kappa_{Q|X_{1}}\left(q|x_{1}^{\prime }\right)P_{X_{1}|X_{1}X_{2}}\left(x_{1}^{\prime }|x_{1},x_{2}\right)\\ P_{Q|X_{1}X_{2}}\left(q|x_{1},x_{2}\right) & =\sum_{x_{2}^{\prime }}\kappa_{Q|X_{2}}\left(q|x_{2}^{\prime }\right)P_{X_{2}|X_{1}X_{2}}\left(x_{2}^{\prime }|x_{1},x_{2}\right). \end{array}$$

We now equate the above two expressions, while using that $P_{X_{1}|X_{1}X_{2}}\left(x_{1}^{\prime }|x_{1},x_{2}\right)=\delta \left(x_{1}^{\prime },x_{1}\right)$ and $P_{X_{2}|X_{1}X_{2}}\left(x_{2}^{\prime }|x_{1},x_{2}\right)=\delta \left(x_{2}^{\prime },x_{2}\right)$ (where δ(·,·) is the Kronecker delta). This gives

(A40) $$\kappa_{Q|X_{1}}\left(q|x_{1}\right)=\kappa_{Q|X_{2}}\left(q|x_{2}\right)$$

for all *q* and any $\left(x_{1},x_{2}\right)$ where $p_{X_{1}X_{2}}\left(x_{1},x_{2}\right)>0$. Now consider a bipartite graph with vertex set $X_{1}\cup X_{2}$ and an edge between vertex $x_{1}$ and vertex $x_{2}$ if $P_{X_{1}X_{2}}\left(x_{1},x_{2}\right)>0$. Define Π to be the set of connected components of this bipartite graph, and let $f_{1}:X_{1}\to \Pi$ be a function that maps each $x_{1}$ to its corresponding connected component (for any $x_{1}$ with $P_{X_{1}}\left(x_{1}\right)=0$, $f_{1}\left(x_{1}\right)$ can be any value). Equation (A40) implies that if $x_{1}$ and $x_{1}^{\prime }$ both belong to the same connected component, then the constraint Equation (A40) will “propagate” from $x_{1}$ to $x_{1}^{\prime }$, so that $\kappa_{Q|X_{1}}\left(q|x_{1}\right)=\kappa_{Q|X_{1}}\left(q|x_{1}^{\prime }\right)$. Said differently, this means that $\kappa_{Q|X_{1}}\left(q|x_{1}\right)=\kappa_{Q|X_{1}}\left(q|f_{1}\left(x_{1}\right)\right)$ and that the Markov condition $\left(X_{1},X_{2}\right)-X_{1}-f_{1}\left(X_{1}\right)-Q$ holds. This gives

(A41) $$I\left(X_{1},X_{2};Q\right)\leq I\left(X_{1},X_{2};f_{1}\left(X_{1}\right)\right)=H\left(f_{1}\left(X_{1}\right)\right),$$

where the first inequality uses the data processing inequality, and the second equality uses that $f_{1}\left(X_{1}\right)$ is a deterministic function of $\left(X_{1},X_{2}\right)$. The upper bound in Equation (A41) is achieved when $Q=f_{1}\left(X_{1}\right)$, thus $Q⊲X_{1}$. A similar argument shows that $Q⊲X_{2}$. We have shown that the constraints in Equation (27) can be replaced by $Q⊲X_{1},Q⊲X_{2}$. It is also clear that any *Q* which is a deterministic function of either $X_{1}$ or $X_{2}$ must also be a deterministic function of the target $Y=(X_{1},X_{2})$, hence I(Y;Q)=H(Q). Combining these results shows that Equation (27) is equivalent to Equation (32) for the COPY gate. □
